# Biometric Recognition: A Systematic Review on Electrocardiogram Data Acquisition Methods

**DOI:** 10.3390/s23031507

**Published:** 2023-01-29

**Authors:** Teresa M. C. Pereira, Raquel C. Conceição, Vitor Sencadas, Raquel Sebastião

**Affiliations:** 1 IEETA, DETI, LASI, Universidade de Aveiro, 3810-193 Aveiro, Portugal; 2 Instituto de Biofísica e Engenharia Biomédica, Faculdade de Ciências da Universidade de Lisboa, Campo Grande, 1749-016 Lisboa, Portugal; 3 Instituto de Materiais (CICECO), Departamento de Materiais e Cerâmica, Universidade de Aveiro, 3810-193 Aveiro, Portugal

**Keywords:** electrocardiogram, biometrics, acquisition methods, acquisition devices, databases

## Abstract

In the last decades, researchers have shown the potential of using Electrocardiogram (ECG) as a biometric trait due to its uniqueness and hidden nature. However, despite the great number of approaches found in the literature, no agreement exists on the most appropriate methodology. This paper presents a systematic review of data acquisition methods, aiming to understand the impact of some variables from the data acquisition protocol of an ECG signal in the biometric identification process. We searched for papers on the subject using Scopus, defining several keywords and restrictions, and found a total of 121 papers. Data acquisition hardware and methods vary widely throughout the literature. We reviewed the intrusiveness of acquisitions, the number of leads used, and the duration of acquisitions. Moreover, by analyzing the literature, we can conclude that the preferable solutions include: (1) the use of off-the-person acquisitions as they bring ECG biometrics closer to viable, unconstrained applications; (2) the use of a one-lead setup; and (3) short-term acquisitions as they required fewer numbers of contact points, making the data acquisition of benefit to user acceptance and allow faster acquisitions, resulting in a user-friendly biometric system. Thus, this paper reviews data acquisition methods, summarizes multiple perspectives, and highlights existing challenges and problems. In contrast, most reviews on ECG-based biometrics focus on feature extraction and classification methods.

## 1. Introduction

Nowadays, recognition systems are used in a variety of real-world applications to protect ourselves and our information. While some of these systems still depend on conventional technologies, such as cards, keys, or passwords, these mechanisms often have usability and security issues.

As a result, there has been a recent interest in the biometric field. Biometric recognition uses distinctive physiological and behavioral characteristics to automatically identify individuals. The former characteristics can include the face, fingerprint, iris, and hand geometry, whereas the latter can be gait signature and keystroke [1].

**Figure 1:**
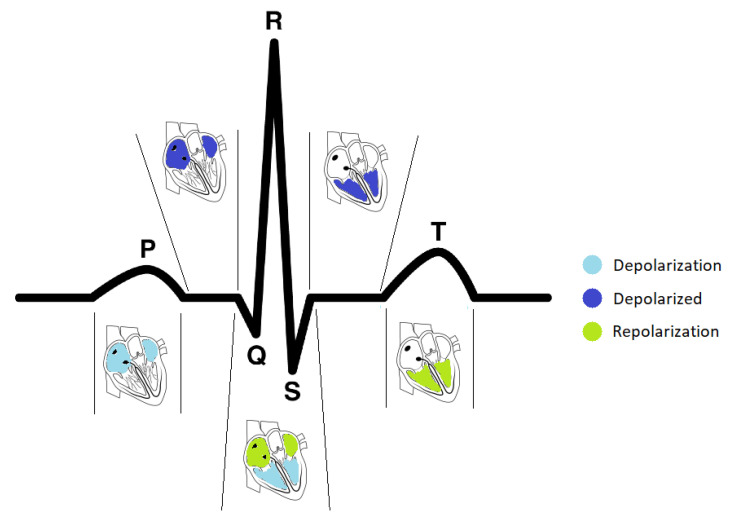
The sequence of depolarization and repolarization events in the heart and their relationship with the different heartbeat waveforms in an ECG signal (adapted from [5], original figure kindly provided by the authors).

In recent years, researchers have been exploring the use of electrocardiogram (ECG) signals as a biometric recognition trait due to their unique properties: (1) liveness detection: Since the ECG is a recording of the electrical activity of the heart, ECG signals can only be acquired from living individuals [2]; (2) high security: ECG signals are extremely difficult to counterfeit and consequently, a technology to artificially produce them has not been developed yet [3]; (3) combined information: the analysis of ECG signals can give us information regarding the identity of a person, as well as heart conditions and emotional and physical status [4]. The most important advantage of ECG signals is their uniqueness among individuals, which is mainly due to changes in ionic potential, the levels of electrolytes in the plasma, and physiological differences caused by chest geometry, size, and position of the heart [2]. A typical ECG wave, such as the one presented in Figure 1, consists of a P wave, a QRS complex, and a T wave.

A biometric system is a technology that identifies or authenticates a person through their unique biometric traits. It consists of three main components: an acquisition module, which consists of a sensor that measures the biometric trait; a storage module, where personal data of enrolled subjects is stored); and a biometric algorithm. The biometric algorithm processes the data from the acquisition and storage modules, following two steps: feature extraction and pattern recognition [5]. Concerning a biometric system using ECG, signals can be acquired through different formats. The standard 12-lead ECG provides information on cardiac activity from 12 different leads over a short period of time, while Holter ECGs record electrical activity from five to seven leads over longer periods of time. Although 12-lead ECGs provide more information, they are not practical for real-world use. Instead, off-the-person methods that acquire ECG signals through skin or finger contact have become more common, making the process more convenient for users [6].

The advancement of sensing technology has made it possible to explore the use of ECG as a non-invasive biometric, similar to a fingerprint. This has made society’s acceptance of ECG as a biometric very promising [2]. In addition to traditional off-the-person methods, small wireless ECG body sensors are being developed for long-term monitoring. These sensors use a single lead to measure the electrical potential difference between electrodes placed near the heart. These sensors allow ECG analysis and monitoring to be used for a wider range of applications beyond diagnosing cardiovascular disorders [6]. However, when compared to medical devices such as Holter devices, wearable sensors produce noisier signals due to various factors, such as the type of electrodes and the number and location of leads. While medical ECG recorders use 12 or 6 wet electrodes, wearable devices typically use between one and three dry electrodes, with only the first lead being used due to its easy implementation in mobile devices. Medical ECG recorders generally provide more reliable data than wearable devices due to their longer and more detailed recording periods and the higher complexity of the setup [7].

In the next stage of the biometric process, features are extracted from the ECG data. These features are specific attributes of the ECG that allow for the recognition of a particular individual based on inter-subject variability. Feature extraction is a crucial step in pattern recognition. Approaches for feature extraction can be divided into three categories: fiducial, non-fiducial, and hybrid (or partially fiducial). Fiducial-based techniques rely on the accurate detection of reference points, such as the P wave, QRS complex, and T wave. These techniques can also use interval, amplitude, angle, and area measurements of these points as biometric features. However, these approaches require a lot of feature engineering, which can be time-consuming [8,9]. Non-fiducial-based ECG biometric detection methods do not require the detection of fiducial points. Instead, non-fiducial features are derived from segmented windows of ECG signals and may include autocorrelation coefficients and wavelet coefficients. Non-fiducial approaches often have a large number of redundant feature sets that need to be reduced [10]. Hybrid methods combine both fiducial and non-fiducial techniques by locating only the R-peaks. These are used to segment the ECG signal into single heartbeat waveforms, from which time or frequency domain information is extracted as features [8].

The final stage of the ECG recognition process consists of classification or pattern recognition. In this stage, the selected features of ECG signals are used as inputs for a classifier. The accuracy of the selection of the features will determine whether the classifier makes a correct or incorrect decision. There are many classification methods that have been proposed in recent years, including Bayesian Network, Linear Discriminant Analysis, Decision Trees, k-Nearest-Neighbors, Support Vector Machines, and Artificial Neural Networks. Each approach has its own advantages and disadvantages [10].

The success of identifying an individual through their ECG depends on the conditions they are exposed to during the acquisition process, the features that are extracted, and the classifiers used for identification or authentication. It is, therefore, important to evaluate the impact that certain changes have on biometric identification results [11]. This systematic review aims to discuss past research on the impact of variables in the data acquisition methods of an ECG signal on the biometric recognition process.

The paper is organized as follows: Section 2 presents the review methodology. Section 3 provides an overview of ECG acquisition and databases, which are discussed in Section 4. Finally, the conclusions drawn are presented in Section 5.

## 2. Review Methodology

In this section, the search strategy, which includes the identification, screening, and inclusion phases, is described, and the research questions we aim to answer are presented.

### 2.1. Search Strategy

**Figure 2:**
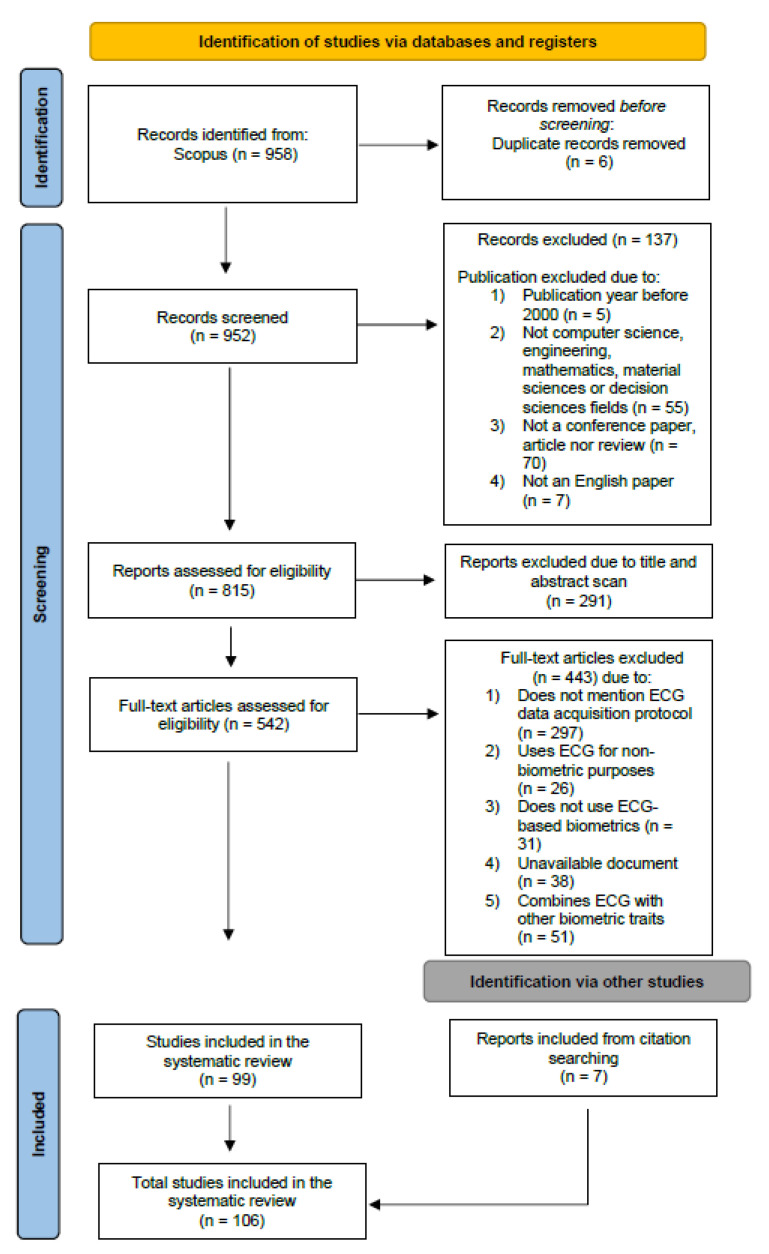
Flow diagram of the literature research process (adapted from Prisma Guidelines [12]).

This systematic review is structured according to Preferred Reporting Items for Systematic Reviews and Meta-Analyses (PRISMA) guidelines [12]. Our literature research was performed from 5 June to 7 October 2022 in the Scopus database. The process of literature search for this literature review is illustrated in Figure 2, and it is a three-stage process consisting of identification, screening, and inclusion.

#### 2.1.1. Identification

For the identification stage, the following general search terms were compiled for the Scopus research on the title, abstract, and keywords fields: (biometric* OR biometry AND ecg* OR electrocardiogram* OR electrocardiography* OR electrocardiographic* OR heart* AND authentication OR identification OR verification OR recognition AND “data collection” OR “signal collection” OR acquisition* OR collection OR signal* OR “body sensors” OR “body sensor” OR sensor* OR biosensor* OR database* OR electrode*). This search resulted in 958 papers. Six of those were duplicates and were consequently removed. Thus, the identification stage resulted in a total of 952 papers.

#### 2.1.2. Screening

Before moving to the manual process of screening, we applied some exclusion criteria in our research. The first criterion concerned the year of publication of the article; only the ones published between 2000 and 2022 were considered. The second criterion was related to the subject area and the following areas were included: computer science, engineering, mathematics, materials science, and decision science. All remaining areas were excluded from our search. The document type was also an exclusion criteria: only conference papers, articles, and reviews the types of paper were considered. Lastly, only papers in English were included. A total of 137 articles were excluded based on the inclusion and exclusion criteria. A total of 815 papers were retrieved from the exclusion criteria process. The second part of the screening stage was a manual process of document exclusion. The purpose of this step was to filter the articles based on their abstract, methodology, results, or findings section to ensure that the articles match the goal of this systematic review. The screening process involved two rounds. In the first round, filtering, and screening were performed to exclude studies based on their respective title and abstract. Studies that did not focus on ECG-based biometric recognition were eliminated in this stage, and a total of 542 articles continued to the following round. The second round performed filtering by an accurate full-text reading of the examined articles from the first round based on an accurate full-text reading. Studies were eliminated based on the following exclusion criteria: (1) not focusing on the data collection process; (2) using ECG for non-biometric purposes; (3) not developing a biometric system algorithm; (4) not available online; and (5) using ECG combined with other biometric traits in a multimodal system. A total of 285 papers were eliminated due to reason (1), seventeen (17) due to reason (2), twenty-eight (28) due to reason (3), thirty-eight (38) due to reason (4), and fifty-three (53) due to reason (5).

#### 2.1.3. Inclusion

**Figure 3:**
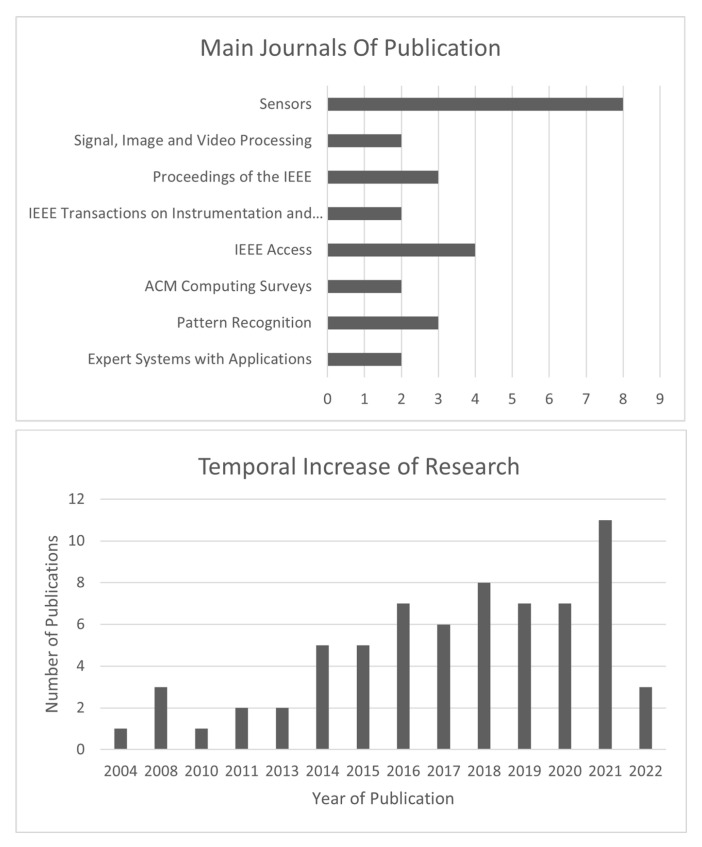
Main journals of publications of the papers included in this systematic review (**top**), and temporal increase of the research on ECG-based biometric systems (**bottom**).

After the screening process, 99 studies were integrated into our search. However, we also added some other reports from citation searching (7), resulting in a total of 106 studies included in this systematic review. The majority of papers included were from the journals/conferences presented in Figure 3 (top). The bottom of this figure presents the temporal increase of research on ECG-based biometric systems.

### 2.2. Research Questions

This work mainly aims to provide some answers to the following questions about the ECG data for biometric systems:

Question 1: How are the ECG signals collected for biometric systems? What is the acquisition hardware information? This review compares the various aspects of the acquisition hardware, such as the intrusiveness of the acquisition (on-the-person vs. off-the-person acquisitions), the number of leads used, and the duration of the acquisition. We also present the most used commercially available and the self-developed acquisition devices and compare them in Table 1.

Question 2: What should the acquisition protocol look like for a biometric system? Which conditions of acquisition should be considered? The aim of the present systematic review is to evaluate and compare the acquisition protocol of different research concerning the number of subjects and the assessment of the stability of the ECG signal over time. Moreover, since the health status of the subjects is also considered by many researchers, this systematic review presents literature findings regarding the impact of physical conditions, posture, emotions, and cardiac conditions on a biometric system.

Question 3: Which ECG datasets are used for biometric purposes? What are the main differences between them? These questions are addressed in Section 3.3 by presenting a description of the most used ECG databases in the literature and by providing a comparison between them in Table 2.

Question 4: Which factors of the data acquisition influence the intra- and inter-subject variability? What impact can these two variables have on the performance of a biometric system? The answers to these questions are discussed in Section 4, in which the sources of intra and inter-subject variability are described.

## 3. ECG Acquisition and Databases

Regarding ECG acquisition, we covered the characteristics of the systems and protocols, as well as commercially available and self-developed devices. Finally, this section presents an overview of the databases used for biometric purposes.

### 3.1. Data Acquisition

Data acquisition can be organized according to criteria, such as the acquisition hardware information and acquisition protocol.

#### 3.1.1. Acquisition Hardware Information

To analyze the characteristics of the acquisition systems, we should consider the intrusiveness of the acquisition and the types of electrodes, the number of leads used, and the duration of the acquisition.

#### Intrusiveness

Since the early research on ECG-based biometrics, the configurations used for data acquisition have significantly evolved. Researchers have mostly focused on addressing the main disadvantage of ECG as a biometric trait: its intrusiveness during data acquisition [5]. This has led to the development of off-the-person data acquisition methods which are less intrusive than traditional medical settings that use multiple wet electrodes. Data acquisition methods can be broadly divided into two categories based on their level of intrusiveness:

1. On-the-Person Acquisitions
  Acquisition methods that require attachment to the body, such as wet Ag/AgCl electrodes applied to the skin with a conductive electrolyte gel, are known as on-the-person methods. This approach relies on half-cell potential, double-layer capacitance, and parallel and series resistances to function. Despite providing good signal quality, wet electrodes can irritate the skin and restrict the user’s movement and may also cause interference between neighboring electrodes. These factors must be considered when using on-the-person data acquisition methods [43].
  This type of acquisition can either be medical or unrestricted by movement, such as through Holter systems. According to medical standards and guidelines, the standard 12-lead configuration allows for the acquisition of an ECG signal in 12 leads (or channels) using three bipolar limb leads, three monopolar limb leads, and six monopolar precordial leads. The orthogonal configuration, also called Frank leads, allows the acquisition of ECG signals using seven electrodes. In early ECG biometric research, recordings from standard 12-lead and Frank leads were used in the development and evaluation of algorithms [44,45,46,47,48,49,50]. Some researchers chose acquisitions without movement restrictions, with longer duration, and with fewer electrodes, such as Holter systems, which can acquire ECG signals for several hours while subjects perform their daily activities [7,51].
2. Off-the-Person Acquisitions
  The off-the-person acquisition method refers to devices that are integrated in objects or surfaces with which the subjects interact (e.g., a computer keyboard) and do not require any special preparation of the subject [52]. Wet electrodes characteristic of medical acquisitions were replaced by dry metallic electrodes, which increase the long-term performance and cause low skin irritation. However, they have high impedance between the electrode and skin, and are susceptible to motion artifacts [43]. Off-the-person acquisitions reduce the number of leads to two or three, and their placements are confined to the upper limbs, especially on the wrists, hands, or fingers [5,15,16,20,24,53,54,55]. Recently, a few initiatives have been conducted to improve off-the-person configurations and approach unconstrained settings in ECG biometrics. These efforts seek to close the gap to real, commercial applications by developing wearable technologies for ECG acquisition or embedding the sensors into common objects [7,11,13,18,28].

Table 3 presents a comparison of on-the-person and off-the-person acquisitions. Some researchers also compared different types of data acquisition and their influence on the performance of the biometric system. Jyotishi et al. [56] evaluated their model using three on-the-person ECG databases and two-off-the-person ECG databases. The results showed that the model performs well for both off-the-person and on-the-person ECG data. Srivastva et al. [2] used two databases, one on-the-person and the other off-the-person, and even mixed them together in a large database. The identification accuracies achieved for both the on-the-person and the off-the-person databases were individually about 99%, whereas an accuracy of approximately 98.5% was obtained for the mixed database. Thus, the authors proved the robustness of their ECG biometric method from signal acquisition methods. Biçakci et al. [7] used data from two different acquisition devices—the wearable-based chest bands and the medical-based Holter—to investigate whether the models are consistent and not biased by device specifications, providing reliable biometric verification with wearable devices. The results achieved for both datasets presented an equal error rate (EER) of around 5% for an enrollment time of 150 s, proving the reliability of using wearable devices for ECG acquisition for biometric purposes. It is also important to note that off-the-person methods have been gaining popularity in recent years for various applications beyond biometric recognition, such as disease detection. For example, in [57], the authors presented a method for recognizing diseases related to ECG and EEG data using sensors available in off-the-shelf mobile devices as well as sensors connected to a BITalino device. This suggests that these types of practical and convenient signal acquisition methods can be useful for a wide range of applications beyond biometric recognition.

#### Number of Leads

**Figure 4:**
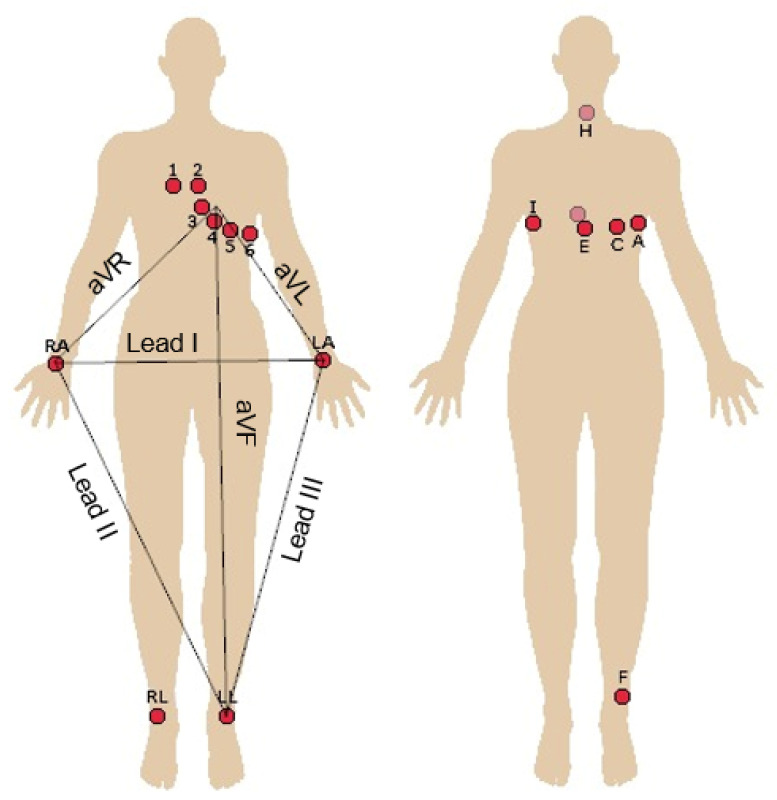
Electrode placement and leads on the standard 12-lead configurations (**left**) and Frank leads (**right**), with the anterior electrodes depicted in red and the posterior electrodes depicted in a lighter red (adapted from [5], original figure kindly provided by the authors).

A standard 12 lead ECG (or even 15-lead ECG) system can record more abnormalities than a single-lead ECG (similar to lead I in a 12 lead ECG). Figure 4 shows a representation of the standard 12-lead and the orthogonal-lead configurations. Due to the practical difficulty of collecting 12-lead ECG, biometric systems with a reduced number of leads have been evaluated.

Dong et al. [44] proposed an identity recognition system and investigated their behavior on the different ECG leads. The experiments based on one-lead ECG showed that the best classification performance was obtained based on lead III and lead V1 and the worst classification performance was obtained based on lead V6. Moreover, experiments based on two-lead ECG outperformed experiments with one-lead ECG. Jekova et al. [59] used 12-lead resting ECG and evaluated the influence of the different leads on a biometric system. The capability of single limb leads was the lowest in III and aVR, and the highest in I and II. The identification capability of single chest leads was the lowest in V3 and the highest in V1. Multi-lead identification models yield considerably higher accuracy (about 20%) compared to the best single-leads. Porée et al. [47] proposed a method testing n = 1, 3, 6, and 12 leads, with all possible n-combinations of 12 leads tested. The best performances were obtained with 12 leads and then decreased with the decrease of the number of leads. With n = 3 and 6, the identification rate (IR) was still greater than 90%, whereas for n = 1, the IR was always lower than 90%. Fang et al. [60] tested their identification system with one- and three-lead ECGs, achieving optimal accuracies of 93% and 99%, respectively. Zhang et al. [9] suggested to place all the ECG electrodes on the left upper-arm or behind the ears in order to achieve excellent wearability. For the acquisition on the arm, the electrodes were integrated into an arm band, whereas for the acquisition behind the ears, electrodes were integrated into headsets or glasses. The signal strength of the single-arm ECG proved to be around 10% of the signal strength of the chest-ECG. However, arm-ECG heartbeats can still be distinguished. Moreover, the ear-ECG was found to be much weaker (5% of chest-ECG), but also shows a great potential for user identification purpose leveraging distinguishable morphologies. The mean accuracy obtained was as high as 98.8% and 91.1% for the single-arm and ear datasets, respectively.

#### Duration of Acquisition

Some researchers also assessed the impact of the duration of the ECG segment on the biometric identification’s performance. In the literature, it is predicted that the shorter the duration of the ECG segment used, the lower the performance obtained by the system. Ramos et al. [11] observed this behavior. However, when increasing the acquisition duration, this conclusion was not valid from a certain point onwards, as more data might introduce redundancy to the system. Results showed that around 10 s of signal are enough to test the identity of an individual. Biçakci et al. [7] evaluated the performance of their proposed method by varying the sample length. They used 5, 50, 150, 250, and 500 s of samples, achieving an EER of around 7% with only 5 s of enrollment. Ibtehaz et al. [61] studied the influence of the number of beats on the performance of the system, and results showed that increasing the number of beats significantly improved the performance. They achieved the perfect 100% accuracy using only three (ECG-ID [32], PTB [36] databases) and six (MIT-BIH Arrhythmia [34] and Normal Sinus Rhythm [35] databases) beats. Bernal-Romero et al. [62] tested their authentication method on different ECG signal duration: 10, 5, and 3 s. The EER rates for the authentication system with ECG signals had average values of 5.99%, 7.12%, and 9.66% for signal lengths of 10 s, 5 s, and 3 s, respectively. Pinto et al. [63] varied the number of enrollment templates between 5, 10, 15, and 30 s, achieving equal error rates of 13.70%, 10.92%, 9.52%, and 7.56%, respectively. Djelouat et al. [64] used testing times from 2 s to 5 s, and results showed an increasing identification rate from 84.44% to 98.88%. Carvalho et al. [23] aimed to measure the minimal number of heartbeats in which it was possible to identify subjects, even in situations where they were under the effect of fear or disgust, using from just one heartbeat up to twenty heartbeats. The results showed an optimal accuracy of around 75–80% when using 5–12 heartbeats.

#### 3.1.2. Acquisition Protocol

#### Time Stability

The temporal separation between biometric evaluations may influence the system’s performance. Chee et al. [65] investigated the influence of different time separations between enrollment and testing data, using PTB [36] and ECG-ID [32] databases, with 83.9 days and 5.5 days between acquisitions, respectively. The model achieved accuracies of 64.16% and 92.70%, for long- and short-time separation, respectively, meaning that the model performance drops significantly when the time separation between the enrollment and classification increases. Ramos et al. [11] studied this influence by evaluating ECG segments from the same/different sessions. The results show that there is a decrease in performance when the sessions are different, except when the signal is collected on the fingers. Thus, the signal acquired on the fingers shows greater stability in the long term. Conversely, the point of acquisition that presents the greatest decrease in performance over time is the chest. Ibtehaz et al. [61] analyzed the cross-session accuracy using two databases, concluding that identification accuracy sharply falls when tested on data from a different session. Nevertheless, an accuracy above 90% was obtained for the ECG-ID database [32] and, for the CYBHi database [30], the accuracy dropped below 80%. Sun et al. [66] proposed a method for biometric identification, reaching a recognition accuracy of about 95%. However, they found that the accuracy degraded dramatically to 40% when considering a significant time interval between the acquisition of the training and testing templates. Porée et al. [47] proposed tests concerning the evaluation of the performances with time, concluding that there may exist a degradation of the ECG stability over time, with performances still acceptable after 16 months.

#### Number of Subjects

The impact of the database size on the accuracy of a biometric system is also a topic of research. Choi et al. [67] used 20, 40, 60, 80, and 100 subjects, reaching an accuracy of 100% for 20 subjects and an accuracy of 96% for 100 subjects, showing that the drop in accuracy when increasing the number of subjects is minimal. Jekova et al. [59] presented consistent validation of their identification models on an independent dataset by increasing its size from 10 to 230 subjects. Their validation results confirmed the expected trend for accuracy drop with the increase in the number of subjects. Chen et al. [53] evaluated their algorithm on 5, 10, 20, 30, 40, and 50 subjects. They noticed that the performance decreases slightly with the number of users increasing (false acceptance rate—FAR—increases from 0.00% to 8.00% and false reject rate—FRR—increases from 0.00% to 10.00% when increasing the number of subjects from 5 to 50). Carreiras et al. [17] tested the recognition system on an ECG signal database with 618 subjects. However, they also tested the system with subsets of this population, assessing the behavior of the recognition system with a varying number of subjects. For the entire database, results showed an EER of 9.01% and an Identification Error (IE) of 15.64%. The results of the population subsets highlight the fact that the EER does not seem to be affected by the population size, while, conversely, the IE increases with the number of subjects.

#### Physical Condition

Some researchers investigated the influence of exercise and body movement on the performance of a biometric system. Ramos et al. [11] studied the impact of the variability caused exclusively by moving the hands, feet, and chest. The results demonstrated that movement of the wrists causes the largest error in biometric identification, followed by movement of the fingers, while movement of the chest has almost no impact on the performance of the method. Huang et al. [68] showed that the ECG signal undergoes small noise interferences while the subject is walking and large noise when the subject is running or jumping. Nobunaga et al. [22] aimed to evaluate the effectiveness of their proposed identification method on exercising humans. They measured ECG during rest for one minute, with the subject lying down, and used these acquisitions to train the model. The exercise ECG used to test the model was measured for each subject after raising their foot so that their heart rate increased to over 100 bpm. The study reached an accuracy of 100% during rest and 99.8% during exercise, indicating that their method is accurate at identifying individuals doing exercise. Komeili et al. [54] also considered the case in which enrollment and testing are in different body conditions: rest and exercise. A feature selection was conducted to select features that are less affected by exercise; these were, then, used for enrolling and testing the biometric system’s users. Experimental results showed an EER of 11%. Moreover, Lee et al. [69] showed that the ECG cycle became shorter after 10 min of physical exercises, running, and holding breath for a certain period.

#### Posture

Most of the studies only consider supine rest conditions, which represent an important limitation regarding the use of ECG-based biometric systems in real-life contexts. Tirado-Martin et al. [21] acquired signals in different posture positions: sitting down at rest, standing at rest, and after exercise. They proved that different heart rates between the enrollment and recognition data result in lower performances. However, the best performance was achieved with the enrollment data acquired in a sitting position at rest. Iqbal et al. [70] achieved an accuracy of 100% when identifying 9 subjects at normal and resting conditions and an accuracy of 96.4% when identifying 39 subjects in 6 different physiological states (working, going up stairs, going down stairs, natural gait, lying with changed position and resting while watching TV). Wahabi et al. [71] considered an enrollment protocol in which each user’s ECG signal is collected under sit, stand, supine, and tripod postures. The accuracies achieved were 98.04% for sit, stand, and supine and 94.12% for tripod. Raj et al. [16] used ECG collected in three postures: a sitting posture at rest, a standing posture at rest, and a sitting posture after 20 s of exercise. They achieved an EER of 4.34% for the “standing” case, whereas the “sitting” and “after-exercise” cases worsened to 11.07% and 12.06%, respectively. Moreover, Wahabi et al. [72] also investigated the effect of body posture on ECG biometric accuracy, demonstrating that the performance of all the methods degraded when the train and testing data were not from the same body position. However, Porée et al. [47] showed that it is still possible to obtain good results even if the position in which the testing data was recorded is not present in the enrolment database.

#### Emotions

An individual’s emotional state is continually changing. These changes occur naturally as a result of body chemistry, levels of stress, and even time of the day. The changes in emotional state are expressed in the ECG trace as changes in heart rate, noise in trace due to muscle flexor action, and variations in electrical potential gain. Thereby, some researchers have investigated the impact of emotions on the identification of individuals [73]. Zhou et al. [74] proposed a method of ECG biometrics using signals acquired under different stress levels, achieving an average recognition rate of 95%. Li et al. [18] used the public database DREAMER, in which the ECG signals acquired from wearable devices are disturbed by the physiological noises from emotional fluctuations induced by different stimuli. The accuracy obtained was of 91.30%, meaning that their method was capable of handling different kinds of emotional disturbances and identifying individuals accurately. Zheng et al. [75] investigated whether ECG based identification was affected by the status of ECG signal collecting, considering four status pairs: emotional status (calm, high pressure), eating (starve, satiation), sleeping (full, lack), and health (healthy, tired/cold). From the obtained results, the authors concluded that negative emotions (high pressure) and lack of sleep reduced the True Positive Rate (TPR) slightly (around 2–3%), but there was no effect on both eating and health pair status. When using ECG data mixed with all four statuses, the overall TPR of identification reached approximately 85%. Zheng et al. [76] self-collected calm and high-pressure ECG datasets to investigate the influence of different emotional statuses. They achieved accuracies of 98.10% and 95.67% for calm and high-pressure data, respectively, showing that the ECG signals under different emotion statuses can be used in reliable and accurate biometric systems. Israel et al. [73] used ECG data collected during seven different tasks performed to stimulate different states of anxiety. The low stress tasks were the subject’s baseline state, mediative, and recovery tasks. The high stress tasks were reading aloud, mathematical manipulation, and driving in virtual reality. Results showed that both within and between anxiety states, nearly all the individuals were correctly classified, as the accuracies obtained were around 97–98%.

#### Cardiac Conditions

The behavior of a biometric system under heart conditions has also been assessed. Chen et al. [77] focused on the comparative performance analysis of human identification with ECG signals collected from subjects in different health conditions. Data used consisted of ECG signals from 38 elderly subjects with a variety of chronic diseases and 30 young healthy students. Experimental results indicated that a better recognition accuracy is achieved for healthy subjects (98.14%) when compared to elderly unhealthy subjects (95.62%). Becerra et al. [78] used a database comprising 20 healthy subjects and 20 pathological subjects (diagnosed with different types of cardiac murmurs). The accuracies obtained were 91.19% and 97.74% for patients with cardiac murmurs and healthy patients, respectively. Singh et al. [79] used the QT database for patients with cardiac diseases and a second database for healthy patients. The proposed ECG biometric method achieved EER of 0.76% and 0.71% in recognizing people suffering from cardiac arrhythmia and people of good health, respectively. Regarding mixed health status, the method achieved an EER of 1.31%, confirming a very good performance and robustness of the proposal. Singh et al. [48] proposed a method to identify arrhythmic and normal subjects, reaching an accuracy of 87.37% for the subjects of MIT-BIH Arrhythmia database [34] and 92.88% for the IIT (BHU) database. Sidek et al. [80] also used three different databases containing various irregular heart states: MIT-BIH Arrhythmia database [34], MIT-BIH supraventricular arrhythmia, and Charles Sturt diabetes complication screening initiative, achieving accuracies of 96.7%, 96.4%, and 99.3% for each, respectively. Loong et al. [81] showed that diseased ECG only reduced the recognition rate by less than 1% and, thus, the system is robust towards diseased ECG. Contrarily, Chiu et al. [82] registered a drop of 19% between identifying normal subjects and subjects with arrhythmia (100% and 81%, respectively). Moreover, Ghazarian et al. [83] assessed the accuracy of ECG-based identification for distinct heart condition groups. They discovered that, in contrast to the initial expectation that identification accuracy for healthy normal sinus rhythm should be the highest, the identification accuracy is higher for patients with sinus tachycardia or patients who are diagnosed with both ST changes and supraventricular tachycardia. Conversely, they observed that patients with premature ventricular contractions have an identification accuracy as low as 78.54% and patients with a pacemaker presented an accuracy of 80.2%.

The conditions under which ECG data are acquired can have a major impact not only on the performance of a biometric system but also on the ability to accurately and reliably detect heart conditions. As such, several studies have been investigating the influence of several factors, such as electrode placement, lead configuration, physical exercise, and the intrusiveness of acquisition, on the detection of heart diseases [84,85,86].

### 3.2. Acquisition Devices

#### 3.2.1. Commercially Available Devices

**Figure 5:**
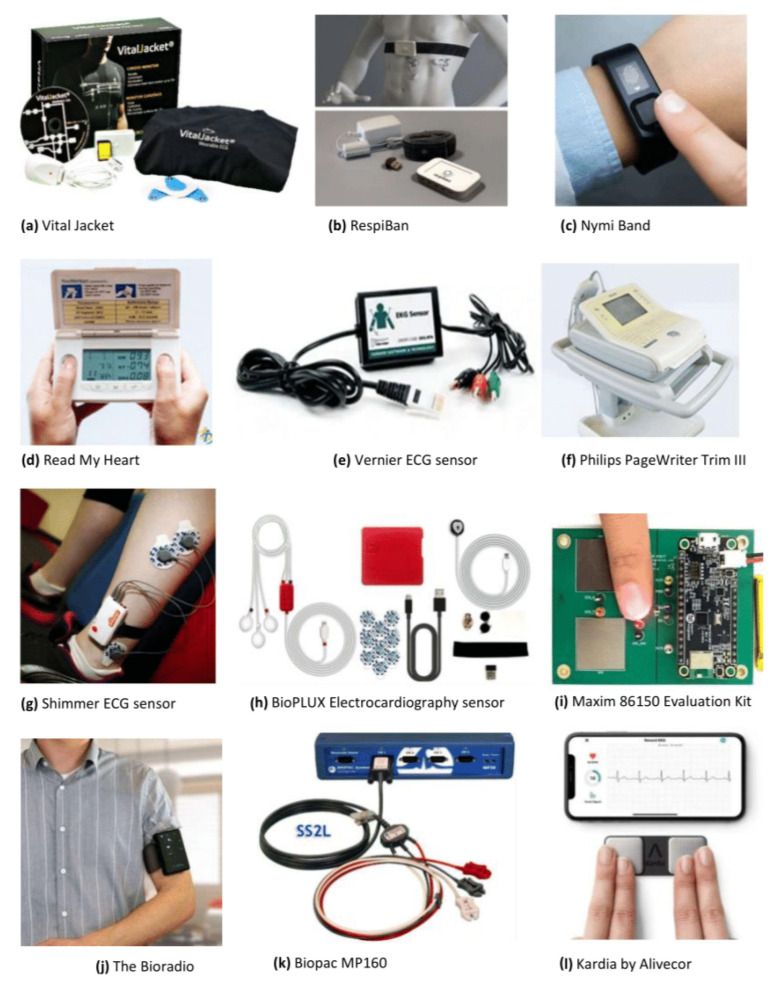
Commercially available devices.

During the last years, the market of medical-grade wearable ECG devices has expanded, and these have increasingly been used for biometric purposes since they can be easily integrated in biometric systems, reducing costs, power consumption, and time of acquisition. However, researchers still use non-wearable devices for data acquisition since they allow records with higher quality. In this section, some of the most used commercially available acquisition devices are presented, as well as some self-developed sensors for ECG acquisitions. Figure 5 shows the commercially available devices described in this section.

1. Vital Jacket
  The VitalJacket [87], presented in Figure 5a, is a wearable device developed by researchers from the IEETA research unit at the University of Aveiro and commercialized by Biodevices SA [88]. It is designed to continuously record high-quality ECG and other vital signals in various clinical and everyday settings. The collected data can be stored on an SD card for offline analysis or transmitted via Bluetooth to mobile devices for real-time monitoring and online processing. Ye et al. [13] investigated the applicability of ECG signals from such wearable device in human identification. In the five-subject study, their proposed method exhibited near 100% recognition rates based on single heartbeats, even with a six-month interval between the training and testing data. Ramos et al. [11] used VitalJacket to collect ECG signals from twenty healthy participants in two sessions separated by 2 weeks. They investigated the impact of movement, the influence of using different ECG acquisition placement, the impact of temporal separation between sessions, and the impact of the acquisition time. The authors reached an accuracy of 99% for signals collected on the fingers in two different sessions. For the various experiments, the results suggested that the ECG signals acquired using VitalJacket can be used as robust biometrics.
2. RespiBan
  The RespiBAN Professional, which is presented in Figure 5b, is a wearable system made by PLUX, which includes a PLUX accelerometer biosensor and biosignal acquisition hardware, as well as a respiration biometric sensor embedded in the chest strap fabric. This device can measure various biosignals, including ECG, electrodermal activity (EDA), electromyogram (EMG), and skin temperature. The collected data can be transmitted to mobile devices via Bluetooth. Biçakci et al. [7] used the WESAD dataset, which consists of ECG recordings collected from a RespiBAN device. The EER obtained was 7.07%, meaning that ECG biometrics will be a valid verification option (or could be in the future) using wearable devices for data acquisition.
3. Nymi Band
  The Nymi Band [89], shown in Figure 5c, is a wearable device that uses the wearer’s unique cardiac signal to unlock Bluetooth-enabled devices such as computers, smartphones, and cards. To authenticate the user, the Nymi Band is placed on the wrists and the top of the device is touched with a finger. As long as the device is worn, the user remains authenticated. The Nymi Band is equipped with a heart rate monitor, accelerometer, gyroscope, and biometric authenticator, and is powered by a rechargeable battery. Chun et al. [14] used ECG data from 15 subjects collected using the Nymi Band, achieving an EER of 0.9%, which proves the reliability of this wearable device.
4. ReadMyHeart
  ReadMyHeart [90], shown in Figure 5d, is a handheld, non-invasive heart monitoring device made by DailyCare BioMedical Inc. It allows users to record electrical signals from their hearts by placing their thumbs on the device’s conducting plates, without the need for wires or conducting gel. The device takes 30 s to record each measurement and displays the average heart rate, ST segment, and QRS interval. These readings are based on a “modified Lead I-ECG”, rather than traditional standard ECG readings. Islam et al. [15] captured ECG signals from 112 individuals using the handheld ECG device ReadMyHeart, achieving a minimum EER of 10.52%.
5. Vernier ECG Sensor
  The Vernier ECG Sensor [91], presented in Figure 5e, is a device used to measure the electrical potential waveforms produced during the contraction of the heart. It can be used to record standard three-lead ECG tracings or surface EMG recordings of muscle contractions in various parts of the body. The device is usually associated with the Vernier Go!Link interface, which is a low-cost USB sensor interface that connects Vernier sensors to a computer. Raj et al. [16] used the Vernier sensor for ECG acquisitions on the arm with different body postures, achieving an Optimal performance with an EER of 4.34%.
6. Philips PageWriter Trim III
  The PageWriter Trim III [92] is a compact and cost-effective cardiograph made by Philips, shown in Figure 5f. It is an interpretative ECG system designed for fast-paced clinical environments, with features such as a high-resolution full-color display and the ability to report, store, and transmit 12-lead ECG data using industry-standard XML. Carreiras et al. [17] used Philips PageWriter Trim III for ECG acquisitions of 618 subjects, achieving an EER and an IE of 9.01% and 15.64%, respectively.
7. Shimmer ECG Sensor
  The Shimmer ECG unit [93] is a device designed for the measurement of physiological signals for ECG, and it is presented in Figure 5g. It includes a configurable digital front-end and an ECG sensor that can record the pathway of electrical impulses through the heart muscle. The sensor can be used to record ECG data on resting and ambulatory subjects, or during exercise to provide information on the heart’s response to physical exertion. Li et al. [18] used a public database, DREAMER, in which data were acquired using the Shimmer ECG Sensor, and an accuracy of 97.2% was obtained.
8. BioPLUX Electrocardiography Sensor
  The BioPLUX [94] low-noise ECG local differential triode configuration enables fast application and unobtrusive single-lead ECG data acquisition. This sensor can be used to extract heart rate data and other ECG features, enabling its application in research fields such as biomedical, biofeedback, psychophysiology, and sports, among many others. Silva et al. [19] used the BioPlux Electrocardiography Sensor presented in Figure 5h integrated on a steering wheel for in-vehicle driver recognition, achieving an IE of 2.40%.
9. Maxim 86150 Evaluation Kit
  Maxim 86150 Evaluation Kit [95], presented in Figure 5i, is a device designed to evaluate the photoplethysmogram (PPG) and ECG bio-sensor module. The device includes a Microcontroller Board and a Sensor Board. The Microcontroller Board houses a microcontroller unit (MCU) with preloaded firmware, Bluetooth connectivity, and power management. The Sensor Board includes the MAX86150 Bio-Sensor Module and two stainless steel dry electrodes for ECG measurement. The Evaluation Kit is powered by an included lithium polymer battery, which is charged with a micro-USB cable. When monitoring is active, the module uses IR Proximity Mode to detect each user’s fingers, and a red LED will turn on when a finger is near the module. Sorvillo et al. [20] used the Maxim 86150 Evaluation Kit to collect ECG for human identification under rest and mental and physical stress, reaching accuracies of 88% and 68%, respectively.
10. The BioRadio
  The BioRadio [96] is a wireless biomedical monitor, shown in Figure 5j, with programmable channels for recording and transmitting various combinations of human physiological signals. It is easy to set up and operate, and the wearable device captures data in a flexible file format compatible with a variety of software suites and proprietary tools. Huang et al. [68] used the BioRadio device with the positions of the electrodes following the Einthoven’s configuration. Abdelazez et al. [97] also used this device, but the electrodes were positioned under the right and left thumbs instead. Their system achieved a precision of 0.68, being able to identify 98.7% of the false positives while retaining the true positives rate.
11. Biopac MP160
  The BIOPAC MP160 [98] is a 16-channel system designed for the acquisition of various physiological signals, including Heart Rate Variability (HRV), Electroencephalogram (EEG), EMG, EGG, and many more. The device, represented in Figure 5k, offers multiple configurations to suit different research and teaching needs, and records multiple channels with different sample rates up to 400 kHz. Used in conjunction with AcqKnowledge software and BIOPAC electrodes, amplifiers, transducers, and other system components, the MP160 is part of a complete data acquisition and analysis system. Many researchers used the BIOPAC system for data acquisition of their proposed biometric system [21,22,23].
12. Kardia by ALIVECOR
  Kardia [99] is a wireless device that allows users to record a medical-grade single-lead ECG in 30 s and receive instant analysis on their phones. It is clinically validated, CE marked, and FDA-cleared, making it a reliable option for checking one’s heart from home. Kardia, in Figure 5l, is compatible with most popular phones and tablets and, to use it, one only needs to download the Kardia app. Arteaga-Falconi et al. [24] used the Kardia device along with a mobile phone for ECG acquisitions at different times and conditions, proving the reliability of this mobile device, since the results revealed 1.41% of FAR and 81.82% of true acceptance rate.

#### 3.2.2. Self-Developed Acquisition Devices

As mentioned above, there are also some researchers who developed their own ECG sensors.

1. Savvy
  Rashkovska et al. [25] developed a wireless ECG sensor for long-term monitoring and tested it in various applications, including biometric authentication. The initial prototype of the wireless body sensor (WBS) was powered by a coin-sized battery and included a low power microcontroller and a 2.4 GHz radio transceiver. The design was later improved to include a rechargeable battery and a Bluetooth Low Power (BLE) radio transceiver for communication. The WBS is attached to the skin by using self-adhesive electrodes and has evolved into a more flexible and lightweight design that allows for unobtrusive long-term health monitoring and low-cost implementation. It is now commercially available as the SavvyTM sensor. The proposed methodology for biometric authentication using this device achieved an EER from 6% to 13%, depending on the subject.
2. Basco et al. [26]
  Blasco et al. [26] developed a wearable sensor capable of measuring photoplethysmography (PPG), ECG, Galvanic Skin Response (GSR), and Acceleration (ACC) signals from the wrists. The ECG sensor is from Bitalino [100] and the two electrodes were placed on the inner side of the wristband and on top of the wristband, respectively. The viability of the use of the sensor on a biometric system was tested in three different acquisition conditions: sitting, walking, and sitting after exercise, and the results were promising.
3. Guven et al. [3]
  Guven et al. [3] also developed a fingertip ECG data acquisition device for biometric purposes. The device consists of two dry-contact sensors, produced by Plessey Semiconductors, an instrumentation amplifier, an anti-aliasing filter, an optocoupler, a digital signals controller (DSC), and a USB connection unit. The authors conducted an experiment to evaluate the performance of the proposed device by comparing it to the use of lead-I ECG signal, recorded using Biopac MP36 with three conventional Ag/AgCl electrodes and gel. The results achieved were around 100% for the IE, showing that this portable, inexpensive, and user-friendly device is very promising for biometric applications.
4. Wieclaw et al. [10]
  Wieclaw et al. [10] developed a sensor using an Arduino Uno and e-Health Sensor Platform V2.0 for data acquisition. Arduino Uno is a microcontroller board with 16 MHz quartz crystal and a USB port for programming, debugging, and data transfer. The e-Health Sensor Platform V2.0 extends the Arduino Uno and enables the implementation of biometric and medical applications. Data acquisition was performed using differential OpAmp schema followed by 8-bit ADC operating at 277 Hz sampling rate. ADC data were transferred to a PC via the COM-port using the PySerial Library. Modified schema required the user to touch the electrodes with two fingers from the left hand and one finger from the right.
5. Peter et al. [27]
  Peter et al. [27] used a low-cost sensor and designed a sensor processing board. They used conventional wet cloth electrodes with repositionable conductive adhesive hydrogel to measure the electrical activity from the skin surface. Then, a sensor board that amplifies and filters the signals was designed. They applied a standard difference amplifier approach, which is a suitable solution since the basic ECG data is obtained as an output of the difference of two leads placed on the body. The circuit build consists of three parts: the differential amplifier, a filter, and a post amplifier. Afterwards, the signal follows to an embedded target platform, called Raspberry Pi (RPi), which is a low-power single-board computer. One advantage of the RPi is that it is supported by MATLAB Simulink and a range of design tools, which facilitates easy and fast prototyping.
6. Ramli et al. [28]
  Ramli et al. [28] developed a portable ECG detection kit integrated into a wearable bracelet that is responsible for detecting the heartbeat signal of the user and sending out the ECG signals to be processed via Bluetooth. The sensor is equipped with three electrodes and by placing a finger on the topside electrode while the user’s wrists are in contact with the other two electrodes, an electrical circuit is completed; ECG signals are able to be detected by the device. The heartbeat detection kit is formed by six main parts: instrumentation amplifier (IA), high-pass filter (HPF), 60 Hz notch filter, low-pass filter (LPF), analog-to-digital converter (ADC), and signal transmitter. They also developed an Android platform application that acts as a secure login system. This application receives the serial data from the heartbeat detection kit through a Bluetooth connection. Then, when the sign-in or sign-up function of the application is triggered, the incoming data is saved to the database. The database will trigger the back-end system which is the Intel platform board to perform the embedding, features extraction, and pattern-matching processes. Once the processes are done, the verification result will be sent to the android application GUI.
7. Lourenço et al. [29]
  Lourenço et al. [29] proposed a method and device for ECG acquisition, using a single lead setup at the fingers, with Ag/AgCl electrodes without gel. This setup aims to increase the usability and acceptability of ECG-based biometric systems to the level of other biometric traits in terms of signal acquisition. The rigid base integrates three leads which, due to the underlying sensor design, correspond to the ground, positive, and negative poles. The right-hand thumb is used as a negative electrode and the left-hand index finger acts simultaneously as the positive and ground electrodes. The base sensor is an ecgPLUX active ECG triode and the transmission was done via a Bluetooth wireless bioPLUX research biosignal acquisition unit.

Table 1 show an overview of the commercially available and self-developed sensors described in Section 3.2.1 and Section 3.2.2.

### 3.3. Databases

Currently, there are several collections publicly available for ECG biometrics research. Below, the most relevant of the currently available ECG collection are characterized. Table 2 summarizes the characteristics of each. Some are publicly available and can be found on physionet [101].

#### 3.3.1. On-the-Person

1. MIT-BIH Arrhythmia Database
  The MIT-BIH Arrhythmia database [34] is a widely used resource for ECG-based biometrics research and is available at the Physionet repository. It consists of 48 half-hour ECG recordings from 47 subjects that were collected in the laboratories at Boston’s Beth Israel Hospital. Out of 27 subjects, 23 recordings were selected from a mixed population of inpatients (about 60%) and outpatients (about 40%), and the remaining 25 recordings were selected from the same set to include less common but clinically significant arrhythmias. The recordings were digitized at 360 samples per second per channel with an 11-bit resolution over a 10 mV range [49].
2. MIT-BIH Normal Sinus Rhythm Database
  This database is composed of excerpts from 18 subjects from the MIT-BIH Arrhythmia database presented above, which are deemed to be free from arrhythmias or other diseases. Subjects included in this database were found to have had no significant arrhythmias, and they include 5 men, aged 26 to 45 and 13 women, aged 20 to 50 [35].
3. MIT-BIH Atrial Fibrillation
  This database contains 25 long-term ECG recordings of human subjects with atrial fibrillation (mostly paroxysmal). The individual recordings are each 10 h in duration and include two ECG signals. The original analog recordings were made at Boston’s Beth Israel Hospital using ambulatory ECG recorders with a typical recording bandwidth of approximately 0.1 Hz to 40 Hz [40].
4. PTB Diagnostic Database
  This database is obtained by the Physikalisch-Technische Bundesanstalt (PTB), National Metrology Institute of Germany [36]. The database contains 549 records with diverse profile information and various lengths of ECG from 290 subjects. Of the 290 subjects, 148 had suffered from myocardial infarction, 18 had cardiomyopathy or heart failure, and 52 were healthy subjects. Acquisitions were performed both through the standard 12-leads and the three Frank leads [49].
5. ECG-ID Database
  The ECG identification database was recorded for biometric identification purposes [32]. Each raw ECG record was acquired for about 20 s with a sampling rate of 500 Hz and a 12-bit resolution. The first two records acquired on the same day were used for each subject. The database consists of 310 one-lead ECG recording sessions obtained from 90 volunteers during a resting state. The number of sessions for each volunteer varied from 2 to 20, with a time span of 1 day to 6 months between the initial and last recordings [49].
6. E-HOL-03-0202-003 Database
  This is an ECG database from the University of Rochester that is focused on biometrics. The study population consists of 202 healthy subjects from the Intercity Digital Electrocardiogram Alliance (IDEAL) database. The database includes 24 Holter recordings that were acquired using the SpaceLab-Burdick digital Holter recorder. The equipment provides 200 Hz sampling frequency signals with 16-bit amplitude resolution. The ECG was acquired using a pseudo-orthogonal lead configuration (X, Y, and Z), obtained through four electrodes placed on the chest. There is an initial resting supine period with a duration of 20 min before starting the ambulatory recording [33].
7. QT Database
  The QT database is a collection of ECGs that have been selected to showcase a wide range of QRS and ST-T shapes, with the goal of testing QT detection algorithms with real-world variability. These records were largely drawn from various ECG databases, including the MIT-BIH Arrhythmia Database [34], as well as additional recordings gathered at Boston’s Beth Israel Deaconess Medical Center. The additional recordings were chosen to represent extreme examples of cardiac (patho)physiology, including data from Holter recordings of patients who experienced sudden cardiac death during the recordings, as well as age- and gender-matched patients without diagnosed cardiac disease. The QT database includes a total of 105 fifteen-minute excerpts of two-channel ECGs [37].
8. Drive Database
  This database contains data collected from a real-world driving task designed to measure a driver’s stress level. The driving protocol involved following a predetermined route for 20 min on open roads in the Boston area while following a set of instructions. Four types of physiological sensors were used during the experiment: ECG, electromyogram (EMG), skin conductivity (EDA and GSR), and respiration. These sensors were connected to a FlexComp analog-to-digital converter, which isolated the subject from the power supply. The ECG electrodes were positioned in a modified lead II configuration to minimize motion artifacts and maximize the amplitude of the R-waves, and the ECG was sampled at 496 Hz. In total, 27 collections were recorded, 6 from drivers who completed the course only once, and 7 from 3 drivers who repeated the course on multiple days [31].
9. Fantasia Database
  The Fantasia Database is a collection of 120 min of continuous ECG recordings taken while subjects were lying down. Two groups of healthy human subjects, ten young and ten elderly participated in this acquisition. Only healthy, nonsmoking subjects with normal exercise tolerance tests, no medical problems, and taking no medication, were admitted to the study. The subjects laids supine for 120 min while continuous ECG signals were collected. All subjects remained in a relaxed state with a normal sinus rhythm while watching the movie “Fantasia” from Disney to help maintain wakefulness [39].

#### 3.3.2. Off-the-Person

1. CYBHI Database
  In this work, Silva et al. [30] presented the CYBHI database which consists of 128 ECG recordings acquired using the off-the-person approach. The ECG signals (2 min long) were recorded simultaneously from both wrists and fingers using dry Ag/AgCl electrodes and electrolycra strips, respectively. These sensors were placed on custom hand-shaped support, and data synchronization was ensured using the syncPLUX synchronization kit. The electrodermal activity data was also collected to provide information about the arousal state of the subject, as the acquisition protocol included both neutral and emotional elicitation tasks. The acquisition protocol consisted of short-term and long-term sessions. Short-term sessions were conducted over 2 days with 65 participants. The participants completed an experimental procedure that was 5 min long, during which they watched a low-arousal video and a high-arousal video (a horror movie trailer). Long-term sessions consisted of 2 data acquisition moments separated by a 3-month period with 63 participants. In both phases, only ECG signals from the fingers were recorded, and in each of the sessions, the subjects were seated for 2 min in a resting position with two fingers on the dry Ag/AgCl electrodes [52].
2. UofTDB
  Pouryayevali et al. [38] collected a large database with 1012 ECG recordings from different people. The acquisition hardware consisted of a pad with dry Ag/AgCl electrodes, positioned so that the left thumb was placed on the positive electrode, whereas the right thumb and right forefinger were placed on the negative and reference electrodes, respectively. According to the acquisition protocol, the ECG recordings were performed in the following conditions: supine, tripod, sit, physical exercise, and stand. The ECG signals were recorded for all the subjects while sitting, but they were collected in supine, tripod, physical exercise, and standing conditions only for 63, 63, 71, and 81 participants, respectively. Regarding the time interval, 72, 65, 54, 47, and 43 out of 1012 subjects participated in 2, 3, 4, 5, and 6 acquisition sessions, respectively. The length of each recording ranged from 2 min to 5 min [52].
3. DREAMER Database
  This database contains two-lead ECG recordings taken during affect elicitation using audio-visual stimuli. The data was collected using eight film clips containing scenes from different films that were designed to elicit a range of emotions. Of these eighteen film clips, two were intended to evoke the following nine emotions: amusement, excitement, happiness, calmness, anger, disgust, fear, sadness, and surprise. The film clips were between 65 and 393 s long. ECG was recorded using a SHIMMER wireless sensor. A total of 25 healthy volunteers aged between 22 and 33 years old participated in the study [41].
4. WESAD Database
  This dataset consists of ECG recordings, along with several other physiological signals, collected from 15 subjects using a RespiBan device. The device was placed around the subject’s chest and recorded ECG using a standard three-lead configuration. After the subjects were equipped with the sensors, a 20-min baseline was recorded (baseline condition), while sitting/standing at a table. Neutral reading material (magazines) was provided. During the amusement condition, the subjects watched a set of eleven funny video clips. The following phase was a stress condition, in which the subjects were exposed to the Trier Social Stress Test (TSST), which consists of a public speaking and mental arithmetic task. The subjects had to deliver a five-minute speech on their personal traits in front of a panel of three people, focusing on strengths and weaknesses. After the speech, the panel asked to subjects to count down from 2023 to 0, in steps of 17, and asked to start over if they made a mistake. Both tasks lasted about 5 min, resulting in a total of ten minutes for the stress condition. The amusement and stress conditions were followed by a meditation, in which subjects followed instructions with their eyes closed while sitting in a comfortable position for seven minutes [42].

## 4. Discussion

Based on the data reported so far, the following issues are discussed: (i) the comparison of the acquisition hardware, (ii) the comparison of the acquisition protocol, (iii) inter-subject variability, and (iv) intra-subjects variability.

### 4.1. Acquisition Hardware

#### 4.1.1. Acquisition Devices

Most databases were collected from medical devices that often have more leads, which makes them more informative. However, the large number of electrodes required, their uncomfortable placement, the limited movement allowed, and the duration of recordings, make it difficult to develop robust biometric systems. Some researchers have attempted to address these issues by using acquisition methods that allow more movement and longer durations using fewer electrodes. One of the most prominent examples was the use of Holter systems, which are designed to continuously acquire ECG signals for several hours while the subjects move and perform daily activities. The Holter monitors are smaller devices compared to standard 12-lead ECG devices, but they still use many wires to connect the electrodes to the recording machine. While these monitors have become more advanced and capable of recording high-quality single or multi-lead ECG, they can still be uncomfortable for patients to wear and can affect the ECG signal’s strength as a biometric trait [25,102].

Despite the potential of off-the-person systems in a practical setting, there are still some challenges that need to be overcome. Off-the-person systems still require the user to hold the electrode or deliberately place the fingers or palms over them. This prevents us to designate them as unconstrained systems, which puts the ECG at a disadvantage over other biometric traits that can already be used for unconstrained recognition. In addition, the use of dry electrodes in farther placement makes the acquisition more vulnerable to interferences, thus affecting the quality of the signal. The efforts on wearable devices have brought ECG biometrics closer to viable, unconstrained applications.

However, the adoption of wearables for biometrics also introduces new challenges [26]. First, wearable devices tend to use cheaper sensors and hardware than traditional biometric systems. Consequently, sensor readings have more noise, and combined with natural variability in the subject’s state, accuracy is more of an issue. Furthermore, wearable devices have limited computational capabilities and must optimize the usage of their resources to maximize battery life, while providing a quick response to biometric challenges. Hence, these issues must be addressed and adequately solved in order to obtain viable commercial ECG biometric systems [3].

#### 4.1.2. Number of Leads

It is known that each ECG lead contains discriminative information regarding the electrical activity of the heart. However, the use of 12-lead ECG is a very unpractical solution for biometric purposes, as it requires the placement of many electrodes. Hence, biometric systems have been evaluated with a reduced number of leads, since it represents interest from a technological point of view. Some researchers, such as Porée et al. [47], tested the use of different combinations of leads (n = 1, 3, 6, and 12 leads) to evaluate the behavior of the system. As expected, they achieved optimal performances with 12 leads and then the performance decreased with the number of leads. However, with only one lead, the performance was still close to 90%. Concerning the use of single-lead ECG, researchers like Jekova et al. [59] used all 12 leads individually as single-lead configurations in order to assess the influence of each lead. They stated that the capability of single limb leads is highest in lead I (and lead II), which justifies the use of lead I in almost all studies in the literature. Moreover, Zhang et al. [9] tried to reach high wearability by placing the electrodes on the upper arm or behind the ears, using an armband and headsets, respectively, proving that, although the strength of the ECG is much lower than the chest-ECG, it still has a great potential for user identification purposes. Thus, more efforts should continue to be made to improve performance while using a single-lead ECG, gathered with minimal intrusiveness, as it is a much more user-friendly approach.

#### 4.1.3. Duration of Acquisition

Short-term ECG data (less than several minutes) and long-term ECG data can complement each other. Short-term ECG data is cheaper and easier to collect. Many cardiac diseases can be detected based on short-term ECG, so such data represent the primary diagnostic tool in outpatient departments. However, long-term ECG can help to detect diseases with intermittent symptoms such as paroxysmal ventricular fibrillation (VF) and atrial fibrillation (AF) [103]. Data acquisition should be a relatively fast process for biometric scenarios. Nevertheless, it is predicted that the shorter the duration of the ECG segment used, the lower the performance obtained by the recognition system. Thereby, many studies assessed the impact of the duration of the ECG segment on the performance of the biometric system, as mentioned above. In general, this behavior was observed in most studies. However, Ramos et al. [11] showed that this pattern may not always be valid from a certain point onwards, as more data can introduce redundancy to the system. While optimal performances were achieved with ECG segments of 10 s in [11] and [62], Djelouat et al. [64] reached an accuracy of 96.66% with only 5 s of acquisition. Thus, it can be suggested that the optimal duration of acquisition may depend on the conditions of acquisition. Taking into account that the duration of the acquisition for a biometric system should be short, the optimally short acquisition time that does not compromise the performance of the system should be investigated.

#### 4.1.4. Sampling Frequency of Acquisition

In general, to ensure that ECG signals are recorded with sufficient detail and resolution, a sampling frequency of at least 500 Hz is commonly used, since it will allow capturing fast changes in ECG signals [104]. When the sampling rate is lower, more information can be lost in the recording and there is a greater change of high-frequency noise being misinterpreted [5]. According to [105], due to the lack of points available in ECG signals, signals with low sampling frequency are usually inefficient for ECG matching purposes when methods such as cross-correlation, percentage root-mean-square deviation, and wavelet distance measurement are used. As such, the low sampling frequencies used in old commercial systems (e.g., 128 Hz) usually need a reinterpolation of data [106]. There are already some studies that developed enhancement techniques to increase the number of samples of a given ECG data from low sampling frequency recordings [107]. Nevertheless, the frequency at which the ECG should be recorded depends on the specific application and the equipment used for recording [104].

In the past, waves with smaller amplitudes were usually ignored since these were almost always caused by noise. However, with the advent of high-resolution ECG technology, it became possible to detect signals as small as 1 μV through the use of signal averaging techniques. This advancement has provided new insights and has shown that techniques to reduce noise are effective in clinical settings. In [106], the authors investigated the effects of the amplitude resolution of ECG acquisition systems on the P-wave analysis. Results proved that at lower bit resolutions, the percentage error is higher than 40%, meaning that almost half of the results are different from the ones obtained with the highest resolution. They concluded that the high resolution used in modern electrocardiographs was suitable for ECG analysis.

There is no standard regarding data acquisition hardware information. Different studies have used various sources, number of leads, and durations of acquisition, making it difficult to compare results between different datasets. However, by analyzing the literature, it can be concluded that the preferable solutions for the acquisition hardware of a biometric system are the following: (1) an off-the-person approach, which is more realistic to a biometric scenario and easily integrated into a biometric system; (2) a one-lead setup, as it is the most practical solution due to the reduction of the number of contact points, making the data acquisition of benefit to user acceptance; (3) a short-term ECG data, since such data would be faster to acquire and to process, resulting in a user-friendly biometric system.

### 4.2. Acquisition Protocol

#### 4.2.1. Number of Subjects

There is an expected trend for a performance drop with an increase in the number of subjects; several studies have confirmed this expectation by testing the system with a different number of subjects and registering a decrease in performance when increasing the number of subjects [53,59]. However, a biometric system should be able to accurately identify and authenticate many subjects. Although the vast majority of studies have tested their models with small databases, Carreiras et al. [52], for example, used a database with 618 subjects, achieving promising results both on authentication and identification (EER of 9.01% and EI of 15.4%). Thus, larger databases, as well as subsets of those databases, should be used in biometric systems to assess their behavior according to the number of subjects considered.

#### 4.2.2. Time Stability

Multi-session ECG authentication, with enrollment and authentication signals captured across two or more different sessions, has become a more relevant problem of late, primarily because of its similarity to real-world use cases. Error rates calculated using multi-session datasets have been reported to be significantly worse than those using single sessions. Two acquisition sessions in a dataset may differ in several different ways, e.g., signals may be captured under different postures or heart-rate, different hydration levels, or may be captured on different days [108]. Performance degradation might arise from the morphological changes in the heartbeats from one session to another due to variations in physical or physiological states of these subjects [45]. Ramos et al. [11] found a decrease in the performance over time, except when the signal is collected on the fingers, with the chest-ECG being the one that obtained the greatest decrease in performance over time. Thus, off-the-person approaches, which are the most user-friendly, may not suffer significant degradation over time.

### 4.3. Sources of Variability

The ECG signals could be affected by three major sources of variability, namely inter-subject and intra-subject variability and artefact and noise, which will be individually described.

#### 4.3.1. Inter-Subject Variability

Inter-subject variability is the variability between ECGs from different individuals. The ECG signal uniqueness can be assumed to be acquired mainly from the uniqueness of DNA, besides other physical factors such as age, ethnicity, and gender, which contribute to the different ECG variations. Since the ECG signal is universal, stable, and unique, the inter-subject variability can be affected by the orientation of heart mass and the conductivity of cardiac muscle. Despite this inter-subject variability, the ECG signal should remain sufficiently stable over time to enable ECG-based biometric authentication. The main reasons for inter-subject variability of ECG morphology are the heart geometry and the individual attributes.

- Heart Geometry: Heart size, cardiac muscle thickness, and the overall shape of the heart dictate the paths the electrical current follows inside the heart, the number of muscle cells that depolarize, and the time it takes to depolarize the whole heart. Athletes, due to their high levels of physical training, commonly have larger hearts with thicker myocardia, which affects the ECG with higher voltages in the QRS complex and results in lower basal heart rates [109,110].
- Individual Attributes: Age, weight, and pregnancy are some of the individual attributes that can cause shifts in the heart position and/or orientation. These shifts will change the orientation of the electrical current conduction vectors along the heart, meaning the electrodes will detect the signal from a different perspective, thus altering the ECG waveform. For instance, the QRS complex amplitude tends to increase from birth to adolescence and then gradually begins to decrease afterward [8]. The authors in [111] also find that the PR interval increases slightly with increasing age. Studies have shown that the amplitude of the S wave in ECG signals is lower in women than in men between the age interval of 18–40 [112]. While gender differences in ECG signal parameters are more evident in young adulthood, they are known to decrease their effect afterward.

#### 4.3.2. Intra-Subject Variability

The intra-subject variability refers to the differences in ECG signals from the same individual or within a single ECG signal. It is important for a biometric modality to have low intra-subject variability, as well as high inter-subject variability and stability over time. There are several sources of intra-individual variability such as chest electrode position and respiration. While the former induces variation between ECGs of the same individual, the latter induces variability within a particular ECG. Moreover, besides these two factors, intra-subject variability may also be induced by many other factors [8]:

- Physical Exercise: The duration of and the intervals between the different deflections of the heartbeats in an ECG signal vary with the heart rate. These changes are especially visible in the interval between the QRS complex and the T wave in situations of tachycardia (higher heart rates) or brachycardia (lower heart rates). Changes in the heart rate caused by physical exercise or meditation can, effectively, affect the electrocardiogram. While Lee at al. [69] showed that the ECG cycle became shorter after 10 min of physical exercise, running, and holding the breath for a certain period on different dates, Komeili et al. [54] also demonstrated that if, during feature selection, one investigates the features that are less affected by physical exercise, one can still achieve great biometric performance while exercising. Lee et al. [69] also investigated the ECG patterns of smokers, finding that there was also a minimal change in the ECG signal before and after smoking.
- Cardiac Conditions: Medical conditions of the heart can also interfere with the dynamics of the electrical pulse conduction and generate variability. In the scope of biometrics, many databases consisting of ECG signals from patients with cardiac conditions have been used. One of the most studied conditions is arrhythmia, which causes wide variations in the heart rate across time. Chiu et al. [82] mentioned that the low accuracies can be justified by unstable QRS-complexes, causing extracted features to change. However, Becerra et al. [78] stated that even though cardiac conditions affect the performance of the system, accuracies can be higher for some classifiers. Moreover, Ghazarian et al. [83] achieved different accuracies for different heart conditions, meaning that feature selection and classification optimization should be performed considering different cardiac conditions.
- Posture: Postures like standing or lying down differ widely on the position and shape of internal organs. The heart is also affected by this, and changes its position in the thorax, and thus its position in reference with the electrode placement, which causes variations in the collected ECG signal. The vast majority of the ECG acquisitions are performed with the subject lying down at rest. However, Porée et al. [47] stated that there is no requirement or advantage to compare only ECG shapes in supine resting conditions. Moreover, Raj et al. [16] presented more accurate performances while standing (which is a more realistic biometric scenario) than while sitting. Furthermore, Wahabi et al. [72] proved that the performance degrades if the training and testing signals are not from the same position, meaning that the enrollment task should be performed in different positions.
- Emotions: The sympathetic and parasympathetic systems of the autonomous nervous system work to increase or reduce the heart rate, respectively. These systems are under the direct influence of psychological states and thus, under stress, fear, and other strong emotions, fatigue, or drowsiness, the heart rate and the ECG signal can be affected. Even though some researchers still achieved good performances with different levels of stress and anxiety (91% and 97% for [18,73], respectively), some others proved a slightly negative impact of emotions on the performance of a biometric system. As mentioned above, Zhang et al. [75] found a reduction of the true positive rate with ECG signals acquired during high pressure and lack of sleeping situations, whereas the eating and health status did not affect the system. Thus, since there is still no consensus on whether emotions (stress, anxiety, levels of sleeping, levels of thirst and eating, etc.) negatively impact the performance of a biometric system or not, researchers should further investigate these conditions within their systems. However, we must note that it is difficult to design an experimental setup that can induce the same emotion in every subject, as different characters, varying moods, and the inability to accurately self-report an emotional experience may significantly affect the outcome of such a study.

#### 4.3.3. Artefacts and Noise

- Electrode Material: The most used electrodes are silver/silver–chloride coated electrodes with well known frequency-band characteristics and temperature stability. However, different materials can have different characteristics affecting the recording: temperature drift can cause variation of the baseline (low frequency oscillations) while frequency-band modifications can cause attenuation of high frequencies [52].
- hlSensor Location: In the biometric scenario, it is common to use single ECG recording with only two electrodes placed in non-standard locations which can lead to different morphology of the ECG recording with respect to the standard recordings [73].
- Power-line Interference: Capacitive coupling with power lines can induce a superimposed (distorted) harmonic signal whose amplitude can obscure the morphological characteristics of ECG: notch filtering is usually used to remove this interference [52].
- Baseline Drift: Respiration causes changes in thorax volume/electrical impedance, therefore causing the isoelectric level to change slowly (in low frequencies ranges): this artefact is usually removed via low pass filtering [52].
- Movement Artefacts: The contact interface between skin/electrode is subjected to electrochemical reactions of ionic chemical species under the electrode inducing half-cell potentials of the order of 1V or less. However, patient movements can change the electrode position and the chemical concentrations of these species can vary leading to different half-cell potentials and therefore different iso-electrical (baseline) levels [52].

## 5. Conclusions

There are several challenges that must be addressed in order to effectively use ECG as a biometric trait. While many studies have been conducted in reviewing the most popular ECG feature sets and in highlighting similarities and differences among features and classification techniques, there is a lack of research on the data acquisition protocol [113], which is the focus of this work. Regarding the population size, the majority of the studies have been conducted on a small population (a few tens of subjects). Therefore, the applicability of ECG biometric recognition on a large scale was not yet proven. Moreover, almost all studies ignored the variability of the ECG during life span (i.e., variability induced by work, ageing, sport activity, etc.); besides, only a few studies considered the applicability of these techniques when subjects suffer from pathological conditions. ECG recognition in pathological subjects is another aspect worth additional investigation. Finally, it must be emphasized that, while guidelines are available for ECG acquisition in the clinical scenario, there is still a lack of standardization on ECG acquisition (number of leads and their positioning, sampling frequency, number of bits, filtering, type of electrodes, etc.) for biometric applications. However, ECG databases for biometric recognition should, ideally, include recordings at a given sampling frequency and condition from the same subjects in different circumstances (e.g., relaxed, during and after physical training) and for a period of several years [114].

Despite the potential of the ECG to be used as one of the main biometric traits, there are still some challenges that need to be solved, especially regarding the acquisition. Quality research is key to addressing the open issues, taking the advantage of current opportunities and proposing increasingly competitive and applicable ECG biometric systems. Furthermore, this research presents a valuable contribution to the field, emphasizing the importance of ECG data acquisition conditions, not only for biometric recognition, but also for other research topics such as disease detection.

## Figures and Tables

**Table 1:** Overview of the commercially available and self-developed devices.

Acquisition Devices	Type of Acquisition	Type of Electrode	Data Transmission	Performance Accuracy
Vital Jacket	Off-the-person (Wearable)	Conductive fabric electrodes	Bluetooth	Recognition Rate 100% [13]
RespiBan	Off-the-person (Wearable)	Pre-gelled electrodes	Bluetooth	Equal Error Rate 7.07% [7]
Nymi Band	Off-the-person (Wearable)	Dry electrodes	Bluetooth	Equal Error Rate: 0.9% [14]
ReadMyHeart	Off-the-person	Conductive Plates	USB connection	Equal Error Rate: 10.52% [15]
Vernier ECG Sensor	On-the-person	Gel electrodes	VernierGo!Link (USB sensor interface)	Equal Error Rate: 4.34% while walking 8.17% while sitting 10.56% after exercise [16]
Philips PageWriter Trim III	On-the-person	Gel electrodes	-	Equal Error Rate: 9.01%
Error of Identification: 15.64% [17]				
Shimmer ECG sensor	On-the-person	Gel electrodes	-	Identification Rate: between 77.25% and 91.30% for different methods [18]
BioPlux Electrocardiography Sensor	Off-the-person	Dry electrodes Electrolycra	-	Identification Error: 1.66% with dry electrodes 5.61% with electrolycra [19]
Maxim 86150 Evaluation Kit	Off-the-person	Stainless steel dry electrodes	Bluetooth	Identification Rate: 97% [20]
The BioRadio	Off-the-person (Wearable)	Dry electrodes	-	-
BioPac	On-the-person	Wet electrodes/ Adhesive disposable Ag/AgCl wet electrodes	-	Equal Error Rate: 2.69% to 4.71% [21] Identification Rate: 100% at rest, 99.8% exercising [22] Identification Rate: 75% to 80% [23]
Kardia	Off-the-person (Wireless)	Dry electrodes	-	True Accept Rate: 81.82% False Acceptance Rate: 1.41% [24]
Savvy	Off-the-person (Wireless)	Self-adhesive electrodes	Bluetooth	Equal Error Rate: 6% to 13% [25]
Basco et al. [26]	Off-the-person (Wearable)	Dry electrodes	Bluetooth	-
Guven et al. [3]	Off-the-person	Dry electrodes	USB connection	Identification Rate: 100%
Wieclaw et al. [10]	Off-the-person	Dry electrodes	USB connection	Identification Rate: 96%
Peter et al. [27]	On-the-person	Wet cloth electrodes with conductive adhesive hydrogel	-	-
Ramli et al. [28]	Off-the-person (Wearable)	Dry electrodes	Bluetooth	Equal Error Rate: 2%
Lourenço et al. [29]	Off-the-person	Dry electrodes	Bluetooth	Identification Rate: 94.3% Equal Error Rate: 10.1%

**Table 2:** Overview of the most used databases in the literature.

Database	OP	NS	Electrode Placement	Leads/ Electrodes	Health Conditions	Activity/ Posture	Sessions	Publicly Available
CYBHi [30]	Yes	128	Palm + Fingers	2/4	None	Reactions triggered by sound and video	Up to two 5-min sessions, 3 months apart	Yes
Drive DB [31]	No	9	Chest	1/-	None	Rest, highway and city driving	50 min to 1.5 h	Yes
ECG-ID [32]	No	90	Wrists	1/-	None	Siting, unrestrained movement	Various 20s recordings per subject over 6 months	Yes
E-Hol 24h [33]	No	203	Chest	3/4	None	Ambulatory recordings	24 h	Yes
MIT-BIH Arrhythmia [34]	No	47	Chest	2/-	Arrythmias	Ambulatory recordings	30 min	Yes
MIT-BIH Normal [35]	No	18	Chest	2/-	None	Ambulatory recordings	30 min	Yes
PTB [36]	No	290	Chest + Limbs	-/15	Various cardiac conditions	At rest only	1–5 per subject, 38.4–104.2 s	Yes
QT [37]	No	105	Chest	-	Various cardiac conditions	Rest and exercise	15 min	Yes
UofTDB [38]	Yes	1019	Fingers	1/2	None	Sit, stand, supine, exercise and tripod	Up to six 2–5 min recordings over 6 months	No
Fantasia [39]	No	40	Chest + Limbs	12	None	Supine at rest	120 min	Yes
MIT-BIH Atrial Fibrillation	Yes	23	Chest + Limbs	12	Atrial Fibrillation [40]	Ambulatory recordings	10 h	Yes
DREAMER [41]	Yes	23	Limbs	1	None	During emotional stimuli	1 h	Yes
WESAD [42]	Yes	17	-	-	None	Sitting, speaking and watching video clips	-	Yes

**Table 3:** Comparison of on-the-person and off-the-person acquisitions (adapted from [58]).

Item	On-the-Person	Off-the-Person
**Type of Electrodes**	Wet electrodes	Dry metallic electrodes
**Number of Leads**	5, 7, or 12 electrodes	2 or 3 electrodes
**Placement of Leads**	Wrists, ankles, chest	Wrists, hands, fingers
**Movement**	Limited	No restriction
**Noise**	Low	High
**Performance**	High	Medium

## Data Availability

Not applicable.

## References

[1] Pereira T.M.C., Conceição R.C., Sebastião R. (2022). Initial Study Using Electrocardiogram for Authentication and Identification. Sensors.

[2] Srivastva R., Singh A., Singh Y.N. (2021). PlexNet: A fast and robust ECG biometric system for human recognition. Inf. Sci..

[3] Guven G., Gurkan H., Guz U. (2018). Biometric identification using fingertip electrocardiogram signals. Signal Image Video Process..

[4] Huang Y., Yang G., Wang K., Liu H., Yin Y. (2022). Robust multi-feature collective non-negative matrix factorization for ECG biometrics. Pattern Recognit..

[5] Pinto J.R., Cardoso J.S., Lourenço A. (2018). Evolution, Current Challenges, and Future Possibilities in ECG Biometrics. IEEE Access.

[6] Merdjanovska E., Rashkovska A. (2022). Comprehensive survey of computational ECG analysis: Databases, methods and applications. Expert Syst. Appl..

[7] Biçakci H.S., Santopietro M., Boakes M., Guest R. Evaluation of Electrocardiogram Biometric Verification Models Based on Short Enrollment Time on Medical and Wearable Recorders. Proceedings of the International Carnahan Conference on Security Technology (ICCST).

[8] Uwaechia A.N., Ramli D.A. (2021). A Comprehensive Survey on ECG Signals as New Biometric Modality for Human Authentication: Recent Advances and Future Challenges. IEEE Access.

[9] Zhang Q., Zhou D. (2018). Deep Arm/Ear-ECG Image Learning for Highly Wearable Biometric Human Identification. Ann. Biomed. Eng..

[10] Wieclaw L., Khoma Y., Falat P., Sabodashko D., Herasymenjo V. Biometric identification from raw ECG signal using deep learning techniques. Proceedings of the 9th IEEE International Conference on Intelligent Data Acquisition and Advanced Computing Systems: Technology and Applications (IDAACS).

[11] Ramos M.S., Carvalho J.M., Pinho A.J., Brás S. (2021). On the Impact of the Data Acquisition protocol on ECG Biometric Identification. Sensors.

[12] PRISMA: Transparent Reporting of Systematic Reviews and Meta-Analyses. https://prisma-statement.org//.

[13] Ye C., Kumar B.V.K.V., Coimbra M.T. Human Identification Based on ECG Signals from Wearable Health Monitoring Devices. Proceedings of the 4th International Symposium on Applied Sciences in Biomedical and Communication Technologies.

[14] Chun S.Y., Hang J.H., Kim H., Lee C., Oakley I., Kim S.P. ECG based user authentication for wearable devices using short time Fourier transform. Proceedings of the 39th International Conference on Telecommunications and Signal Processing (TSP).

[15] Islam M.S., Alajlan N. (2017). Biometric template extraction from a heartbeat signal captured from fingers. Multimed. Tools Appl..

[16] Raj P.S., Hatzinakos D. Feasibility of single-arm single-lead ECG biometrics. Proceedings of the 22nd European Signal Processing Conference (EUSIPCO).

[17] Carreiras C., Lourenço A., Silva H.P., Fred A.L.N. (2016). Evaluating Template Uniqueness in ECG Biometrics. Informatics in Control, Automation and Robotics.

[18] Li W., Zhang Z., Hou B., Song A. (2021). Collaborative-Set Measurement for ECG-Based Human Identification. IEEE Trans. Instrum. Meas..

[19] Silva H., Lourenço A., Fred A. In-vehicle driver recognition based on hand ECG signals. Proceedings of the 2012 ACM International Conference on Intelligent User Interfaces.

[20] Sorvillo R., Bacco L., Merone M., Zompanti A., Santonic M., Pennazza G., Iannello G. Single beat ECG-based Identification System: Development and robustness test in different working conditions. Proceedings of the IEEE International Workshop on Metrology for Industry 4.0 & IoT.

[21] Tirado-Martin P., Liu-Jimenez J., Sanchez-Cosanova J., Sanchez-Reillo R. (2020). QRS Differentiation to improve ECG Biometrics under Different Physical Scenarios Using Multilayer Perceptron. Appl. Sci..

[22] Nobunaga T., Watanabe T., Tanaka H. (2018). Identification of Exercising Individuals Based on Features Extracted from ECG Frequency Spectrums. IEICE Trans. Fundam. Electron. Commun. Comput. Sci..

[23] Carvalho J.M., Bras S., Ferreira J., Soares S., Pinho A.J. (2017). Impact of the Acquisition Time on ECG Compression-Based Biometric Identification Systems. Pattern Recognition and Image Analysis.

[24] Arteaga-Falconi J.S., Al Osman H., El Saddik A. (2016). ECG Authentication for Mobile Devices. IEEE Trans. Instrum. Meas..

[25] Rashkovska A., Depolli M., Tomasic I., Avbelj V., Trobec R. (2020). Medica-Grade ECG Sensor for Long-Term Monitoring. Sensors.

[26] Blasco J., Peris-Lopez P. (2018). On the feasibility of low-cost wearable sensors for multi-modal biometric verification. Sensors.

[27] Peter S., Reddy B.P., Momtaz F., Givargis T. (2016). Design of Secure ECG-Based Biometric Authentication in Body Area Sensor Networks. Sensors.

[28] Ramli D.A., Hooi M.Y., Chee K.J. (2016). Development of Heartbeat Detection Kit for Biometric Authentication System. Procedia Comput. Sci..

[29] Lourenco A., Silva H.P., Fred A. (2011). Unveiling the Biometric Potential of Finger-Based ECG Signals. Comput. Intell. Neurosci..

[30] Silva H.P., Lourenco A., Fread A., Raposo N., Aires-de-Sousa M. (2013). Check Your Biosignals Here: A new dataset for off-the-person ECG biometrics. Comput. Methods Programs Biomed..

[31] Healey J.A., Picard R.W. (2005). Detecting stress during real-world driving tasks using physiological sensors. IEEE Trans. Intell. Transp. Syst..

[32] Lugovaya T.S. (2005). Biometric Human Identification Based on Electrocardiogram. Master’s Thesis.

[33] E-hol-03-0202-003, University of Rocher Medical Center, Telemetric and Holter ECG Warehouse. http://thew-project.org/database/e-hol-03-0202-003.html.

[34] Moddy G.B., Mark R.G. (2001). The impact of the MIT-BIH Arrhythmia Database. IEEE Eng. Med. Biol..

[35] Goldberger A., Amaral L., Glass L., Hausdorff J., Ivanov P.C., Mark R., Stangley H.E. (2000). PhysioBank, PhysioToolkit, and PhysioneNet: Components of a new research resource for complex physiologic signals. Circulation.

[36] Bousseljot R., Kreiseler D., Schnabel A. (1995). Nutzung der EKG-Signaldatenbank CARDIODAT der PTB über das Internet. Biomed. Tech..

[37] Laguna P., Mark R.G., Goldberger A.L., Moody G.B. (1997). A Database for Evaluation of Algorithms for Measurement of QT and Other Waveform Intervals in the ECG. Comput. Cardiol..

[38] Pouryayevali S. (2015). ECG Biometrics: New Algorithm and Multimodal Biometric System. University of Toronto (Canada) [ProQuest Dissertations Publishing 1604768]. https://www.proquest.com/openview/5c65393cd4e46efa7af666f4ed901773/1?pq-origsite=gscholar&cbl=18750.

[39] Iyengar N., Peng C.K., Morin R., Goldberger A.L., Lipsitz L.A. (1996). Age-related alterations in the fractal scaling of cardiac interbeat interval dynamics. Am. J. Physiol..

[40] Moddy G.B., Mark R.G. (1983). A new method for detecting atrial fibrillation using R-R intervals. Comput. Cardiol..

[41] Katsigiannis S., Ramzan N. (2018). DREAMER: A Database for Emotion Recongition Through EEG and ECG Signals from Wireless Low-cost Off-the-Shelf Devices. IEEE J. Biomed. Health Inform..

[42] Schmidt P., Reiss A., Duerichen R., Marberger C., Laerhoven K.V. Introducing WESAD, a Multimodal Dataset for Wearable Stress and Affect Detection. Proceedings of the 20th ACM International Conference on Multimodal Interaction (ICMI’18).

[43] Rathore A.S., Li Z., Zhu W., Jin Z., Xu W. (2021). A survey on Heart Biometrics. ACM Comput. Surv..

[44] Dong X., Si W., Yu W. (2020). Identity Recognition Based on the QRS Complex Dynamics of Electrocardiogram. IEEE Access.

[45] Wu S., Chen P., Hsieh J. (2018). Spatialtemporal features of electrocardiogram for biometric recognition. Multidimens. Syst. Signal Process..

[46] Dalal S., Vishwakarma P., Sisaudia V. ECG Classification using Kernel Extreme Learning Machine. Proceedings of the 2nd IEEE International Conference on Power Electronics, Intelligent Control and Energy Systems (ICPEICES).

[47] Porée F., Kervio G., Carrault G. (2016). ECG biometric analysis in different physiological recording conditions. Signal Image Video Process..

[48] Singh Y.N., Singh S.K. (2013). Human Identification Using Heartbeat Interval Features and ECG Morphology. Proceedings of the Seventh International Conference on Bio-Inspired Computing: Theories and Applications (BIC-TA 2012).

[49] Ingale M., Cordeiro R., Thentu S., Park Y., Karimian N. (2020). ECG Biometric Authentication: A Comparative Analysis. IEEE Access.

[50] Agrafioti F., Hatzinakos D. Fusiono f ECG sources for human identification. Proceedings of the 3rd International Symposium on Communications, Control and Signal Processing.

[51] Labati R.D., Sassi R., Scotti F. ECG Biometric recognition: Permanence analysis of QRS signals for 24 h continuous authentication. Proceedings of the IEEE International Workshop on Information Forensics and Security.

[52] Merone M., Soda P., Sansone M., Sansone C. (2017). ECG databases for biometric systems: A systematic review. Expert Syst. Appl..

[53] Chen Y., Chen W. Finger ECG-based authentication for healthcare data security using artificial neural network. Proceedings of the IEEE 19th International Conference on e-Health Networking, Applications and Services (Healthcom).

[54] Komeili M., Louis W., Armanfard N., Hatzinakos D. On evaluating human recognition using electrocardiogram signals: From rest to exercise. Proceedings of the IEEE Canadian Conference on Electrical and Computer Engineering (CCECE).

[55] Lin S.L., Chen C.K., Lin C.L., Yang W.C., Chiang C.T. (2014). Individual identification based on chaotic electrocardiogram signals during muscular exercise. IET Biom..

[56] Jyotishi D., Dandapat S. (2022). An ECG Biometric System Using Hierarchical LSTM With Attention Mechanism. IEEE Sens. J..

[57] Ponciano V., Pires I.M., Ribeiro F.R., Villasana M.V., Teixeira M.C., Zdravevski E. (2020). Experimental Study for Determining the Parameters Required for Detecting ECG and EEG Related Diseases during the Timed-Up and Go Test. Computers.

[58] Shdefat A., Mostafa N., Saker L., Topcu A. (2021). A survey study of the current challenges and opportunities of deploying the ECG biometric authentication method in IoT and 5G environments. Indones. J. Electr. Eng. Inform..

[59] Jekova I., Krasteva V., Schmid R. (2018). Human Identification by Cross-Correlation and Pattern Matching of Personalized Heartbeat: Influence of ECG Leads and Reference Database Size. Sensors.

[60] Fang S.C., Chan H.L. (2009). Human identification by quantifying similarity and dissimilarity in electrocardiogram phase space. Pattern Recognit..

[61] Ibtehaz N., Chowdhury M.E.H., Khandakar A., Kiranyaz S., Rahman M.S., Tahir A., Qiblawey Y., Rahman T. (2022). EDITH: ECG Biometrics Aided by Deep Learning for Reliable Individual Authentication. IEEE Trans. Emerg. Top. Comput. Intell..

[62] Romero J.C.B., Echeverri J.M.R., Cortés J.M.R., Gil P.G., Magdaleno J.R., Vega I.C. On signal variability of ECG-based biometric system under practical considerations. Proceedings of the IEEE Mexican Humanitarian Technology Conference.

[63] Pinto J.R., Cardoso J.S. An End-to-End Convolutional Neural Network for ECG-Based Biometric Authentication. Proceedings of the IEEE 10th International Conference on Biometrics Theory, Applications and Systems (BTAS).

[64] Djelouat H., Disi M.A., Amira A., Bensaali F., Zhai X. (2018). Compressive Sensing Based ECG Biometric System. Intelligent Systems and Applications.

[65] Chee K.J., Ramli D.A. (2022). Electrocardiogram Biometric Using Transformer’s Self-Attention Mechanism for Sequence Pair Feature Extractor and Flexible Enrollment Scope Identification. Sensors.

[66] Sun H., Guo Y., Chen B., Chen Y. A Practical Cross-Domain ECG Biometric Identification Method. Proceedings of the 2019 IEEE Global Communications Conference (GLOBECOM).

[67] Choi G.H., Bak E.S., Pan S.B. (2019). User Identification System Using 2D Resized Spectrogram Features of ECG. IEEE Access.

[68] Huang P., Guo L., Li M., Fang Y. (2019). Practical Privacy-Preserving ECG-Based Authentication for IoT-Based Healthcare. IEEE Internet Things J..

[69] Lee W., Kim S., Kim D. (2018). Individual Biometric Identification Using Multi-Cycle Electrocardiographic Waveform Patterns. Sensors.

[70] Iqbal F.T.Z., Sidek K.A. Cardioid graph based ECG biometric using compressed QRS complex. Proceedings of the International Conference on BioSignal Analysis, Processing and Systems (ICBAPS).

[71] Wahabi S., Pouryayevali S., Hatzinakos D. Posture-invariant ECG recognition with posture detection. Proceedings of the IEEE International Conference on Acoustics, Speech and Signal Processing (ICASSP).

[72] Wahabi S., Pouryayevali S., Hari S., Hatzinakos D. (2014). On evaluating ECG Biometric Systems: Session-Dependence and Body Posture. IEEE Trans. Inf. Forensics Secur..

[73] Israel S., Irvine J., Cheng A., Wiederhold M., Wiederhold B. (2005). ECG to identify individuals. Pattern Recognit..

[74] Zhou R., Wang C., Zhang P., Chen X., Du L., Wang P., Zhao Z., Du M., Fang Z. (2021). ECG-based biometric under different psychological stress states. Comput. Methods Programs Biomed..

[75] Zheng G., Zhang H.Y., Ji S.Z. ECG based identification under different physical status In Proceedings of the International Conference on Machine Learning and Cybernetics (ICMLC).

[76] Zheng G., Ji S., Dai M., Sun Y. (2017). ECG based identification by deep learning. Chin. Conf. Biom. Recognit..

[77] Chen M., Li Y.F., Bao S.D., Zhang Y.J. A comparative performance study of electrocardiogram-based human identity recognition. Proceedings of the IEEE International Conference on Computational Science and Engineering (CSE) and IEEE International Conference on Embedded and Ubiquitous Computing (EUC).

[78] Becerra M., Duque-Mejía C., Zapata H.J., Peluffo D., Serna-Guarín L., Delgado-Trejos E., Revelo J., Blanco X. (2018). Exploratory Study of the Effects of Cardiac Murmurs on Electrocardiographic-Signal-Based Biometric Systems. Lect. Notes Comput. Sci..

[79] Singh Y.N. (2015). Human recognition using Fisher’s discriminant analysis of heartbeat interval features and ECG morphology. Neurocomputing.

[80] Sidek K.A., Khalil I., Jelinek H.F. (2014). ECG Biometric with Abnormal Cardiac Conditions in Remote Monitoring System. IEEE Trans. Syst. Man, Cybern. Syst..

[81] Loong J.L.C., Swee S.K., Bear R., Subari K.S., Abdullah M.K. Effects of diseased ECG on the robustness of ECG biometric systems. Proceedings of the IEEE EMBS Conference on Biomedical Engineering and Sciences (IECBES).

[82] Chiu C.C., Chuang C.M., Hsu C.Y. (2009). Discrete Wavelet Transform Applied on Personal Identity Verification with ECG Signal. Int. J. Wavelets Multiresolution Inf. Process..

[83] Ghazarian A., Zheng J., El-Askary H., Chu H., Fu G., Rakovski C. Increased Risks of Re-Identification For Patients Posed by Deep Learning-Based ECG Identification Algorithms. Proceedings of the 43rd Annual International Conference of the IEEE Engineering in Medicine & Biology Society (EMBC).

[84] Tison G.H., Zhang J., Delling F.N., Deo R.C. (2019). Automated and Interpretable Patient ECG Profiles for Disease Detection, Tracking, and Discovery. Circ Cardiovasc. Qual Outcomes.

[85] Rath A., Mishra D., Panda G., Satapathy S.C. (2021). Heart disease detection using deep learning methods from imbalanced ECG samples. Biomed. Signal Process. Control..

[86] Karaoğuz M.R., Yurtseven E., Aslan G., Deliormanlı B.G., Adıgüzel O., Gönen M., Li K.M., Yılmaz E.N. (2019). The quality of ECG data acquisition, and diagnostic performance of a novel adhesive patch for ambulatory cardiac rhythm monitoring in arrhythmia detection. J. Electrocardiol..

[87] Cunha J.P.S., Cunha B., Pereira A.S., Xavier W., Ferreira N., Meireles L. Vital-Jacket: A wearable wireless vital signs monitor for patients’ mobility in cardiology and sports. Proceedings of the 2010 4th International Conference on Pervasive Computing Technologies for Healthcare.

[88] VitalJacket Holter—Biodevices. https://www.vitaljacket.com/pt/vitaljacket-holter/.

[89] Nymi Band. https://www.nymi.com/.

[90] Daily Care ReadyMyHeart ECG Recorder. https://ekgshop.com/product/daily-care-readmyheart-ecg-recorder/.

[91] EKG Sensor - Vernier Science Education. https://www.vernier.com/product/ekg-sensor/.

[92] Philips PageWriter Trim III EKG - SOMA TECH INTL. https://www.somatechnology.com/EKG-Machines/Philips-Pagewriter-Trim-III.aspx.

[93] Shimmer3 ECG Unit. https://shimmersensing.com/product/shimmer3-ecg-unit-2/.

[94] Electrocardiography (ECG) Sensor. https://www.pluxbiosignals.com/products/electrocardiography-ecg-sensor-1.

[95] Maxim Integrated MAX86150 Evaluation Kit (MAX86150EVSYS#). https://eu.mouser.com/new/maxim-integrated/maxim-max86150-eval-kit/.

[96] The BioRadio—Wireless Biomedical Monitor. https://glntdevelopment.com/bioradio/bioradio-wireless-physiological-monitor/.

[97] Abdelazez M., Hozayn M., Hanna G.S.K., Chan A.D.C. Gating of false identifications in electrocardiogram based biometric system. Proceedings of the IEEE International Symposium on Medical Measurements and Applications (MeMeA).

[98] MP160 STARTER SYSTEMS. https://www.biopac.com/product-category/research/systems/mp150-starter-systems/.

[99] KardiaMobile EKG Monitor by Alivecor. https://store.kardia.com/products/kardiamobile.

[100] HeartBIT PLUX. https://www.pluxbiosignals.com/collections/bitalino/products/heartbit.

[101] Physionet Database. https://physionet.org/about/database/.

[102] Blaco J., Chen T.M., Tapiador J., Peris-Lopez P. (2017). A survey of Wearable Biometric Recognition Systems. ACM Comput. Surv..

[103] Hong S., Zhou Y., Shang J., Xiao C., Sun J. (2020). Opportunities and challenges of deep learning methods for electrocardiogram data: A systematic review. Comput. Biol. Med..

[104] Sörnmo L., Laguna P. (2005). Bioelectrical Signal Processing in Cardiac and Neurological Application, Biomedical Engineering.

[105] Chan A.D.C., Hamdy M.M., Badre A., Badee V. (2008). Wavelet distance measure for person identification using electrocardiograms. IEEE Trans. Instrum. Meas..

[106] Censi F., Calcagnini G., Corazza I., Mattei E., Triventi M., Bartolini P., Boriani G. (2022). On the resolution of ECG acquisition systems for the reliable analysis of the P-Wave. Physiol Meas..

[107] Sidek K.A., Khalil I. (2013). Enhancement of low sampling frequency recordings for ECG biometric matching using interpolation. Comput. Methods Programs Biomed..

[108] Ranjan A. Permanence of ECG Biometric: Experiments Using Convolutional Neural Networks. Proceedings of the International Conference on Biometrics (ICB).

[109] Hoekema R., Uijen G.J.H., Oosterom A.V. (1999). Geometrical aspects of the inter-individual variability of multilead ECG recordings. Comput. Cardiol..

[110] Oosterom A.V., Hoekema R., Uijen G.J. (2000). Geometrical factors affecting the interindividual variability of the ECG and the VCG. J. Electrocardiol..

[111] Aro A.L., Anttonen O., Kerola T., Junttila M.J., Tikkanen J.T., Rissanen H.A., Reunanen A., Huikuri H.V. (2014). Prognostic significance of prolonger PR interval in the general population. Eur. Heart J..

[112] Macfarlane P.W., Lawrie T.D.V., Macfarlane P.W., van Oosterom A., Pahlm O., Kligfield P., Janse M., Camm J. (2010). The Normal Electrocardiogram and Vectorcardiogram. Comprehensive Electrocardiology.

[113] Sansone M., Fusco R., Pepino A., Sansone C. (2013). Electrocardiogram Pattern Recognition and Analysis Based on Artificial Neural Networks and Support Vector Machines: A review. J. Healthc. Eng..

[114] Fratini A., Sansone M., Bifulco P., Cesarelli M. (2015). Individual identification via electrocardiogram analysis. Biomed. Eng. Online.

